# A systematic review on silica-, carbon-, and magnetic materials-supported copper species as efficient heterogeneous nanocatalysts in “click” reactions

**DOI:** 10.3762/bjoc.16.52

**Published:** 2020-04-01

**Authors:** Pezhman Shiri, Jasem Aboonajmi

**Affiliations:** 1 Department of Chemistry, Shiraz University, Shiraz, Iran

**Keywords:** “click” reaction, copper complexes, reusable catalysts, Sharpless–Meldal C–N bond-forming reaction, silica/carbon/magnetic materials

## Abstract

In recent years, many inorganic silica/carbon-based and magnetic materials have been selected to arrest copper ions through a widespread range of anchoring and embedding methodologies. These inorganic supported nanocatalysts have been found to be efficient, environmentally friendly, recyclable, and durable. In addition, one of the vital issues for expanding new, stable, and reusable catalysts is the discovery of unique catalysts. The basis and foundation of this review article is to consider the recently published developments (2014–2019) in the synthesis and catalytic applications of copper supported by silica nanocomposites, carbon nanocomposites, and magnetic nanocomposites for expanding the “click” chemistry.

## Introduction

Heterocycles are a class of organic compounds with signiﬁcant biological activities [1–10], including antimicrobial [2], antibacterial [3], anti-HIV [4], antiviral [5], antiparkinsonian [6], and anticancer [10] properties.

Particularly, triazoles illustrate distinguished moieties well distributed in natural products with biological properties [2–4,9–10], including antimicrobial [2], antibacterial [3], antifungal [3], anti-HIV [4], and anticancer [10] activities.

One of the employed procedures for the creation of triazole products is the Huisgen azide–alkyne cycloaddition, and the reaction selectively forms one type of triazole products. Many of the alkyne and azide substrates are commercially available, many others can easily be prepared with a good range of functional groups. The intramolecular reaction of an alkyne as a dipolarophile with an azide as a 1,3-dipole to produce the desired 1,2,3-triazole motif is a model of “click” chemistry. The concept of “click” chemistry is an idiom that was developed by Sharpless and Meldal and later by others to describe organic reactions that are stereospecific, high-yielding, modular, wide in scope, executable under simple reaction conditions, conductable in safe or easily removable solvents, and that generate only nonoffensive byproducts. Of course, the classical Huisgen 1,3-dipolar cycloaddition does not fit into the above definition and fails as a real “click” reaction. Although this cyclization reaction requires elevated temperatures and often yields both the 1,4- and 1,5-regioisomers, the Cu or Ru alkyne–azide cycloaddition falls exactly into the above definition [11].

In this respect, the copper-catalyzed cycloaddition reaction proceeds under mild conditions, is effective, efficient, and requires no column purification in many cases. The Cu alkyne–azide cycloaddition (CuAAC) version also gives only 1,2,3-triazole products substituted at the 1- and 4-positions in an aqueous medium even at room temperature and requires no protecting groups [12]. Later, ruthenium complexes-catalyzed alkyne–azide cycloadditions (RuAACs) regioselectively produced the opposite form of the disubstituted triazoles. Thus, a wide range of azides was reacted with diverse nonactivated terminal alkyne substrates using ruthenium complexes to generate 1,2,3-triazole products substituted at the 1- and 5-positions [13].

A range of copper(I) species (copper(I) iodide, copper(I) bromide, [Cu(CH_3_CN)_4_]PF_6_, (EtO)_3_P⋅CuI, and [Cu(PPh_3_)_3_]Br) has been applied in the prementioned reaction [14]. Generally, Cu(I) species are not thermodynamically stable and can be oxidized to Cu(II) species. Without using a ligand or a reducing agent, Cu(II) can oxidize alkynes to produce an undesired byproduct. Active copper catalysts can be prepared by reducing copper(II) sources, oxidizing copper metal, comproportionation of Cu(II) and Cu(0), or combination of copper salts and suitable ligands [14].

Ligands serve to increase and modulate the reactivity of copper salts. In the first attempt, tris((1-benzyl-1*H*-1,2,3-triazol-4-yl)methyl)amine (TBTA) considerably speds up the copper-catalyzed cyclization [15]. Many structurally diverse ligands, such as nitrogen-, phosphorus-, carbon-, oxygen-, and sulfur-containing ligands were investigated soon after the disclosure of the auxiliary effect of ligands on copper-catalyzed alkyne–azide cycloaddition reactions [16–17].

Heterogeneous catalysts are safer than their homogeneous counterparts. They also offer some advantages, such as the ease of product separation, catalyst recovery, simplifying the production process, and cleaner operation conditions [18–20].

Thus far, several heterogeneous catalysts have been explored for CuAAC and RuAAC processes. The catalytic activities of heterogeneous copper catalysts as novel catalysts are well-recognized with respect to their benefits, such as easy work-up, simple separation of catalysts from products, and economically viable usage in laboratory processes [21–23].

A new and systematic review of the recently published procedures for constructing copper supported by silica nanocomposite, carbon nanocomposite, and magnetic nanocomposites and their catalytic uses in CuAACs will be helpful to the community of scientists working in industry and scholarly laboratories, respectively.

This review aims to cover recently published reports from 2014 to 2019 presenting synthetic routes and applications that focused on the title catalysts in CuAAC reactions.

## Review

### Copper anchored on functionalized silica materials: efficient and recyclable catalysts for CuAAC reactions

In recent years, silica or silicon dioxide nanomaterials have received much attention from researchers and industry and have been used in a wide spectrum of applications. Silica nanomaterials have the empirical formula SiO_2_ in which one silicon atom covalently links to four oxygen atoms, and most of the oxygen bond to two silicon atoms. Hence, their atoms are randomly oriented to produce amorphous structures. These materials, with a high surface area, could be employed as electronic devices, thermal or electronic insulators, catalysts, and humidity sensors [24].

Moreover, mesoporous silica nanomaterials benefit from unique properties, including a large surface area, uniform pore size and distribution, high chemical/thermal/mechanical stability, and good adsorption capacity. They also contain an ordered porous network that is appropriate for the free diffusion of reactants and reaction products [24].

In this regard, various functionalized SiO_2_ nanoparticles were employed in diverse organic reactions, including Suzuki–Miyaura reactions, Ugi–Smiles reactions, Mizoroki–Heck reactions, aldol reactions, oxidation and dehydration reactions, Mannich reactions, and multicomponent reactions. Because of the mentioned reasons as well as due to the low toxicity of silica nanoparticles, they are often a good option [24]. For this review article, we have provided an overview on the synthesis and functionalization of SiO_2_ nanoparticles and on the investigation on their catalytic applications in CuAAC reactions.

**Scheme 1:**
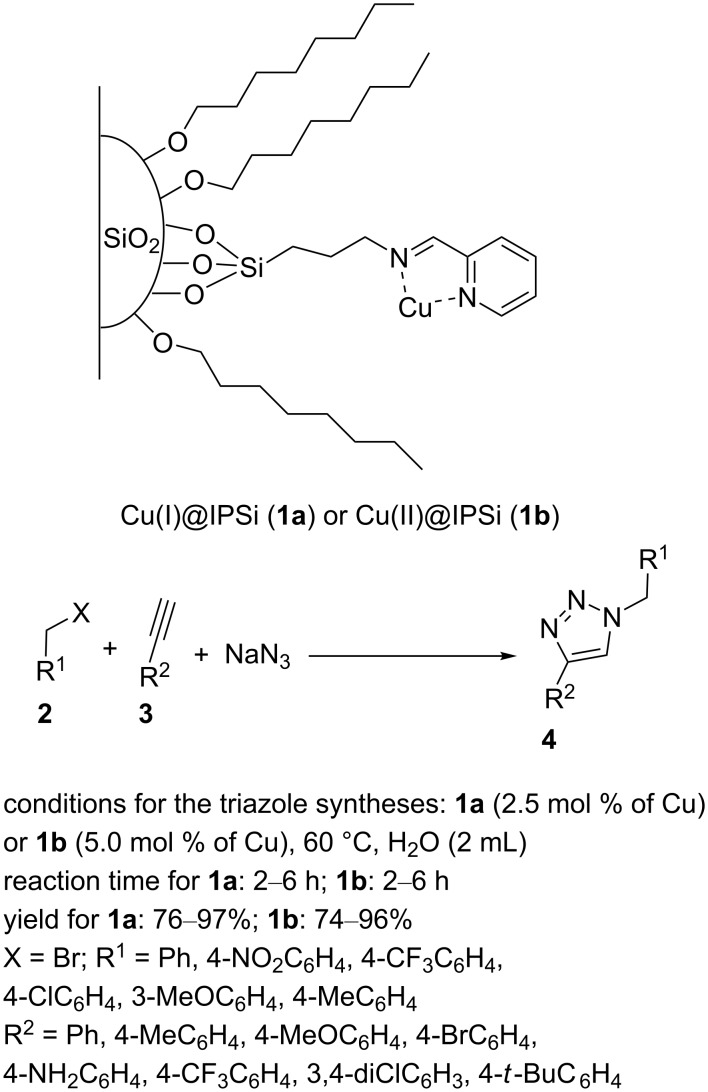
Chemical structure of the catalysts **1a** and **1b** and their catalytic application in CuAAC reactions.

Rhee et al. immobilized Cu(I) and Cu(II) species onto reverse-phased silica gel [25]. In this work, a 2-pyridinecarboxaldehyde ligand was attached on reversed-phase 3-aminopropyl-functionalized silica gel (APSi) in dichloromethane as a solvent. The complexation reaction of the resulting Schiff base, named iminopropyl-functionalized silica gel (IPSi), with [Cu(CH_3_CN)_4_]PF_6_ and CuSO_4_ produced Cu(I)@IPSi (**1a**) and Cu(II)@IPSi (**1b**), respectively (Scheme 1). The catalysts were characterized by a series of analytical techniques. The solid-state ^13^C NMR of IPSi showed nine peaks at 10, 24, 43, 63, 121, 136, 148, 155, and 162 ppm, respectively. SEM analysis displayed an irregular shape for **1a** and **1b**.

The catalytic activity of Cu(I)@IPSi (**1a**) and Cu(II)@IPSi (**1b**) was investigated in the one-pot three-component condensation of various benzyl bromides, various phenylacetylenes, and sodium azide (Scheme 1). The authors stated that benzyl azide was formed in situ by the nucleophilic addition of the benzyl bromide and sodium azide. Subsequently, the desired triazole products were generated through the [3 + 2] cycloaddition of the azide and alkyne. The recyclability analysis of these IPSi-supported Cu(I) and Cu(II) catalysts indicated seven consecutive runs with almost equivalent performances.

**Scheme 2:**
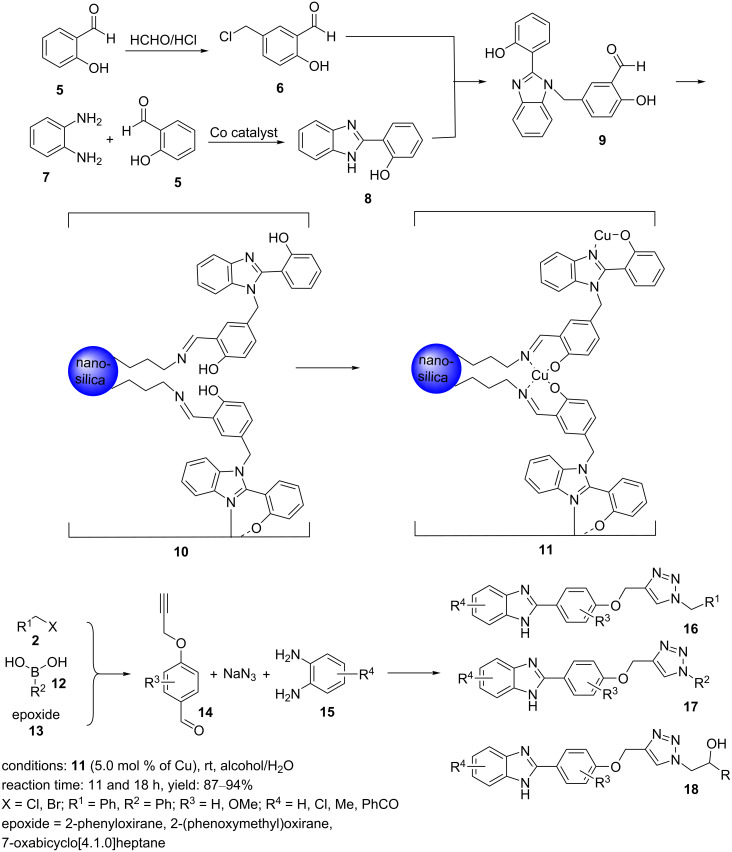
Synthetic route to the catalyst **11** and its catalytic application in CuAAC reactions.

In 2018, silica modified with a benzimidazole–salen Cu(II) complex, **11**, was synthesized in several steps [26]. Initially, 5‐(chloromethyl)‐2‐hydroxybenzaldehyde (**6**) was prepared by the reaction of 2‐hydroxybenzaldehyde (**5**) and paraformaldehyde in concentrated HCl at a temperature between 5 and 10 °C. At the same time, benzimidazole-substituted phenol **8** was generated by the reaction of 2-hydroxybenzaldehyde (**5**) and *o*‐phenylenediamine (**7**) at rt using a cobalt catalyst. In the next step, a benzimidazole-containing aldehyde **9** was obtained by the reaction of **6** with benzimidazole-substituted phenol **8**. This ligand was immobilized on propylamine-functionalized nanosilica. Finally, the complexation reaction of Cu(II) with a supported ligand on propylamine-functionalized nanosilica, **10**, was performed to produce a Cu(II) benzimidazole–salen complex supported by imine-functionalized silica (BS–Cu(II)@SiO_2_ (**11**), Scheme 2).

The nanocatalyst **11** was used to synthesize benzimidazole–triazole derivatives through a one‐pot sequence of activation of the alkyne, [3 + 2] condensation, cyclization, and aromatization under mild reaction conditions (Scheme 2). Moreover, the BS–Cu(II)@SiO_2_ (**11**) catalyst could be recovered and recycled for seven cycles without a notable loss of catalytic activity.

**Scheme 3:**
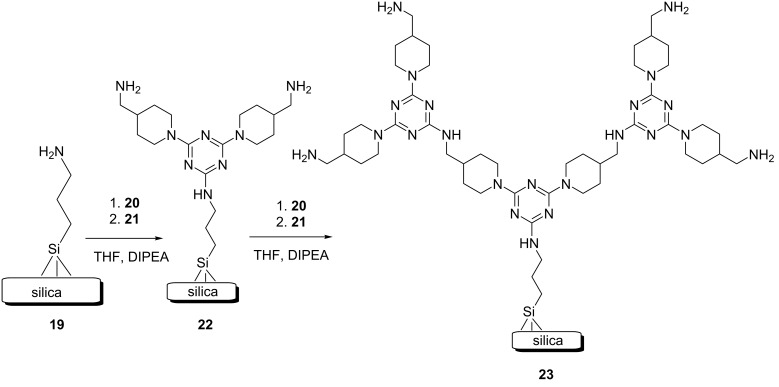
Synthetic route of dendrons, illustrated using G_2_-AMP **23**.

Using a multistep solid-phase procedure, Shantz et al. reported on SBA-15 functionalized with melamine-based dendrons (Scheme 3) [27]. The Cu and Cu/Au nanoparticles were then supported on this new composite to generate a nanocatalyst. The catalytic application of this nanocatalyst was investigated as well. The amine-functionalized SBA-15, **19**, was added to a THF solution of cyanuric chloride and DIPEA. After being stirred in a closed vessel at rt for 24 h, the produced material was ﬁltered off and then washed with methanol, dichloromethane, and THF. The sample was again reacted with 4-aminomethylpiperidine (AMP, **21**) or piperazine (PIP) to afford G_1_-AAA–SBA-15 (G_1_-AMP–SBA-15, **22**, or G_2_-PIP–SBA-15, **23**) materials (Scheme 3). The resulting solid product was then filtrated and washed with methanol, dichloromethane, and tetrahydrofuran. The organic part of the G_1_-AAA–SBA-15 was grown one or two times by repeating the above-mentioned process to generate G_2_-AAA–SBA-15 and G_3_-AAA–SBA-15 composites, respectively. Generally, x and AAA in G_x_-AAA–SBA-15 refer to the dendron generation and diamine (AMP for dendrons containing 4-aminomethylpiperidine and PIP for dendrons containing piperazine), respectively. In the next step, G_x_-AAA–SBA-15 was dispersed in an aqueous solution of HAuCl_4_ and stirred for a short time. The reduction of Au was accomplished using an aqueous solution of NaBH_4_. The obtained material was then centrifuged and washed with deionized water. The resulting solid was dispersed in an aqueous solution of CuCl_2_⋅3H_2_O for 10 min. The copper species was reduced with an aqueous solution of NaBH_4_. The metal–dendron SBA-15 material was ﬁltrated, washed with deionized water, and dried to produce x wt % Cu_Y_Au–G_x_-AAA–SBA-15 (x wt % = weight percent loading of Cu, Y= Cu/Au molar ratio, G_x_ = dendrimer generation, and AAA = dendron type).

**Scheme 4:**
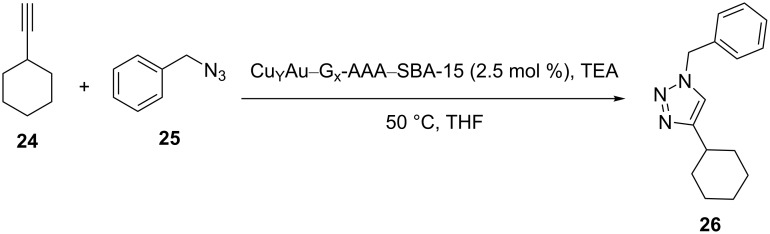
The catalytic application of Cu_Y_Au–G_x_-AAA–SBA-15 in a CuAAC reaction.

After the preparation and characterization of the Cu_Y_Au–G_x_-AAA–SBA-15 catalyst, the material was employed in a triazole synthesis, and it was established that the catalyst was efficient in the Sharpless–Meldal C–N bond formation reaction in Scheme 4.

Different ratios of Cu/Au were explored for the reaction. As expected, the SBA-15, G_2_-AMP–SBA-15, and 2 wt % Au–G_2_-SBA-15 materials were not catalytically active in the reactions. However, the reactions proceeded well using 2 wt % Cu–SBA-15 and 2 wt % Cu–G_2_-AMP–SBA-15 materials.

Different ratios of Cu/Au (1:1, 3:1, and 5:1) were studied for the coupling reaction of a nonactivated terminal alkyne with an organic azide. Control measurements showed that 1:1 and 3:1 samples were more efficient than a 5:1 sample. The authors stated that increasing the Cu/Au ratio caused the formation of thicker layers of Cu on Au nanoparticles. Other findings in this study were: a) copper leaching decreased using gold nanoparticles, b) increasing the dendron generation increased the diffusion resistance.

**Scheme 5:**
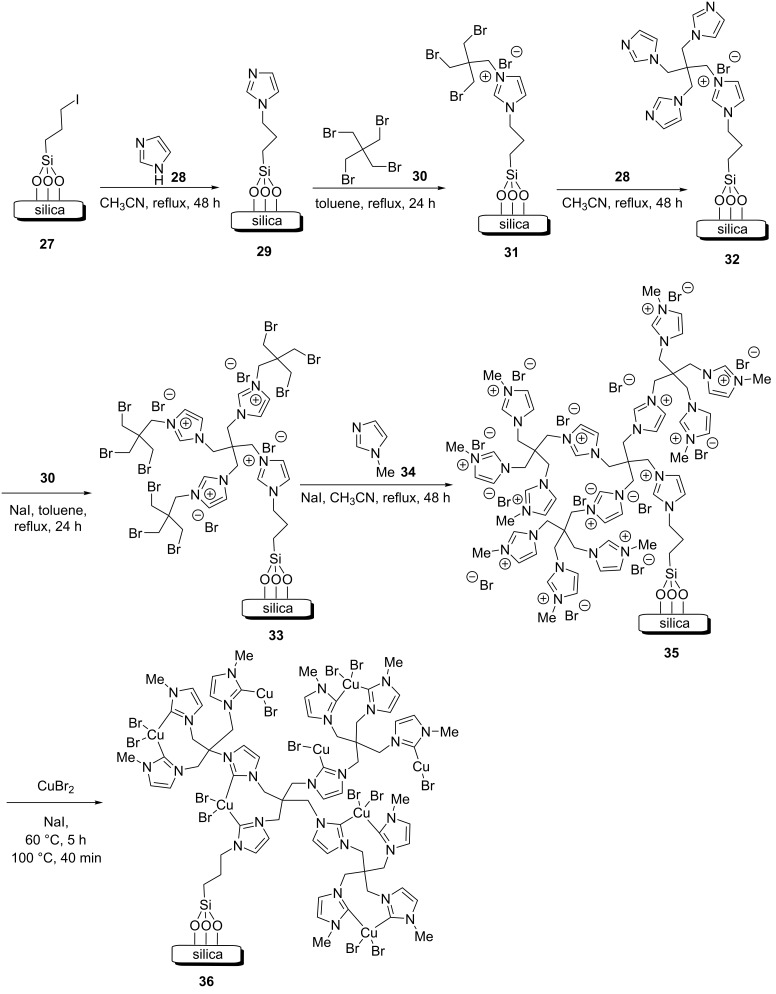
Synthetic route to the catalyst **36**.

As shown in Scheme 5, a silica nanomaterial was decorated with a novel copper complex of poly(N-heterocyclic carbene) in multiple steps [28]. Initially, imidazole (**28**) was immobilized on the silica nanomaterial **27** by a nucleophilic attack of **28** on the iodopropylated silica nanomaterial **27** in acetonitrile under reflux for 48 h. The obtained 1-propylimidazole–nanosilica compound ImP–nSiO_2_ (**29**) was filtered, washed with acetonitrile, and dried. The catalyst **29** was treated with pentaerythritol tetrabromide (PETB, **30**) using NaI in anhydrous toluene under reflux for 24 h. The obtained composite PETB–ImP–nSiO_2_ (**31**) was filtrated, washed with anhydrous toluene, and dried. Then, **28**, **31**, and NaI were added to acetonitrile, and this was stirred under reflux for 48 h. The obtained nanomaterial G_1_–nSiO_2_ (**32**) was filtrated, washed with acetonitrile, and dried. Then, **32** was reacted with **30** and NaI in dry toluene as a solvent at 120 °C for 24 h to yield the nanomaterial PETB–G_1_–nSiO_2_ (**33**). The solid product was gathered by filtration and then dried. The material G_2_–nSiO_2_ (**35**) was prepared by the nucleophilic attack of 1-methylimidazole (**34**) to PETB–G_1_–nSiO_2_ (**33**) in refluxing acetonitrile for 48 h. G_2_–nSiO_2_ (**35**) was then filtrated, washed with acetonitrile, and dried. Cu(II) was immobilized on **35** by stirring in acetonitrile at 60 °C for 5 h and then at 100 °C for 40 minutes. The obtained poly(N-heterocyclic carbine–Cu complex) immobilized on nanosilica, (Cu(II)–NHCs)_n_@nSiO_2_ (**36**), was filtrated, washed with acetonitrile/methanol, and dried.

**Scheme 6:**
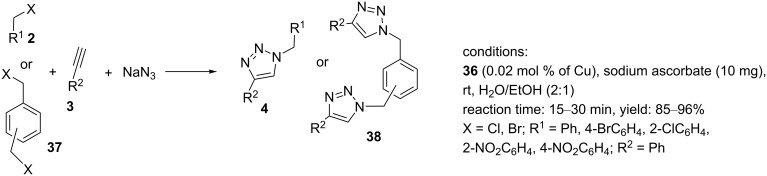
Application of the catalyst **36** in CuAAC reactions.

The catalyst (Cu(II)–NHCs)_n_@nSiO_2_ catalyst (**36**) showed outstanding activity and selectivity, and mono- and bistriazole products were formed using a very low amount of the catalyst **36** and sodium ascorbate in a mixture of water and ethanol as the solvent system at room temperature, with good to high yields being achieved within short reaction times (Scheme 6). Furthermore, compound **36** could be recycled and used in seven subsequent cycles.

**Scheme 7:**
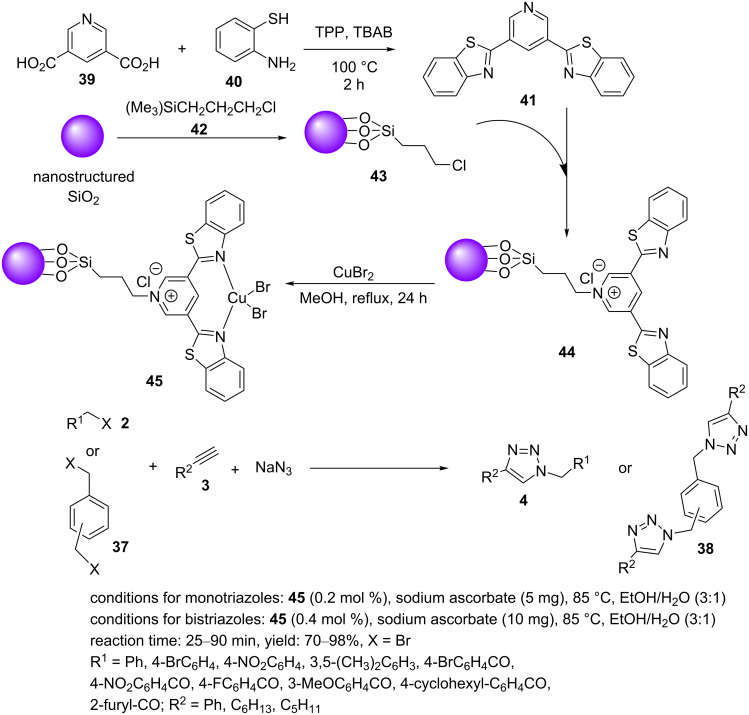
The synthetic route to the catalyst **45** and catalytic application of **45** in “click” reactions.

A further example of a silica-supported CuAAC catalyst was reported by Moghadam et al. (Scheme 7) [29]. In this study, the bis(benzothiazole)-substituted pyridine ligand BTP (**41**) was synthesized through the condensation of 2-aminothiophenol (**40**) with pyridine-3,5-dicarboxylic acid (**39**) using P(OC₆H₅)₃ (TPP). The ligand **41** was immobilized on 3-chloropropyltrimethoxysilane (CPTMS, **43**) to afford TMSP–nSiO_2_ (**44**). Material **44** was then reacted with CuBr_2_ in methanol at reflux for 24 h to produce the heterogeneous catalyst Cu(II)Br_2_–BTP@TMSP–nSiO_2_ (**45**, Scheme 7). X-ray photoelectron spectroscopy (XPS) analysis displayed a Cu 2p_3/2_ peak of the Cu(II)Br_2_–BTP@TMSP–nSiO_2_ catalyst at 933.5 eV attributed to a Cu(II) species.

Monotriazoles were obtained using 0.2 mol % of Cu(II)Br_2_–BTP@TMSP–nSiO_2_ (**45**) and a catalytic amount of sodium ascorbate as a reducing agent in a mixture of water and ethanol as the solvent at 85 °C. In continuation, bistriazoles have been produced using 0.4 mol % of **45** and 10 mg sodium ascorbate as a reducing agent in an ethanol/water mixture at 85 °C. All triazole products were produced in good to high yield (Scheme 7). The ICP analysis of **45** revealed a strong attachment of the copper species to the functionalized nanosilica. Furthermore, the composite **45** demonstrated good catalytic activity in five consecutive cycles.

**Scheme 8:**
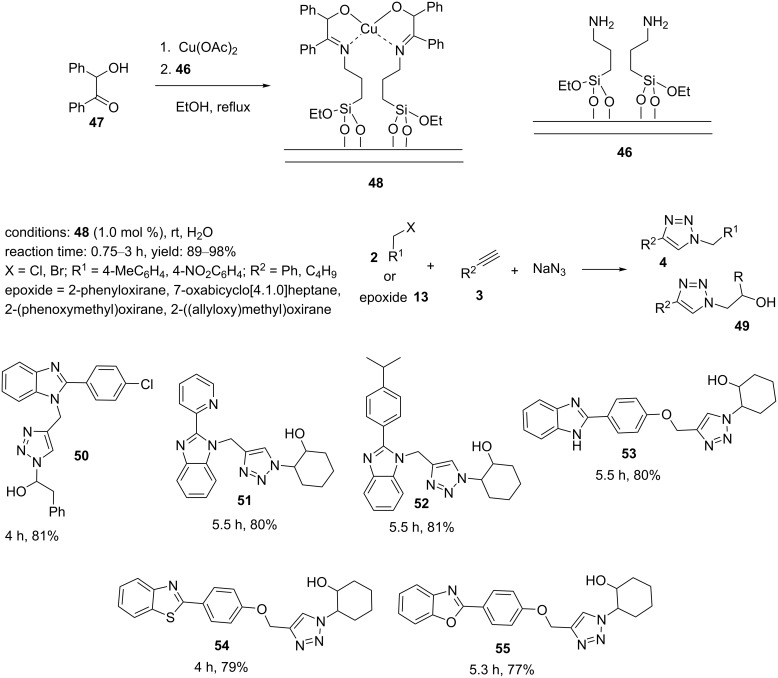
Synthetic route to the catalyst **48** and catalytic application of **48** in “click” reactions.

Recently, the synthesis and catalytic application of a copper(II)–2-imino-1,2-diphenylethan-1-ol complex supported on nanosilica (Cu(II)–ID@silica, **48**) was described by our research group [30]: The silica-supported complex **48** was obtained in two steps (Scheme 8). First, 2-hydroxy-1,2-diphenylethan-1-one (**47**) and copper acetate were stirred and heated to reflux. In the next step, the amino-functionalized silica **46** was added to the mixture, and this was heated to reflux to generate the catalyst **48**. Several organic halides or epoxides, nonactivated alkynes, and sodium azide were reacted in an aqueous medium to provide triazoles or β-hydroxytriazoles using 1.0 mol % catalyst loading at 25 °C. Subsequently, a range of hybrid molecules, including triazole–benzimidazoles **50**–**53**, triazole–benzothiazole **54**, and triazole–benzoxazole **55**, was prepared under the above-mentioned conditions (Scheme 8). The benefits of this catalytic system were mild reaction conditions, low catalyst loadings, a diverse set of triazole products, excellent reusability, short reaction times, and good to high yields.

**Scheme 9:**
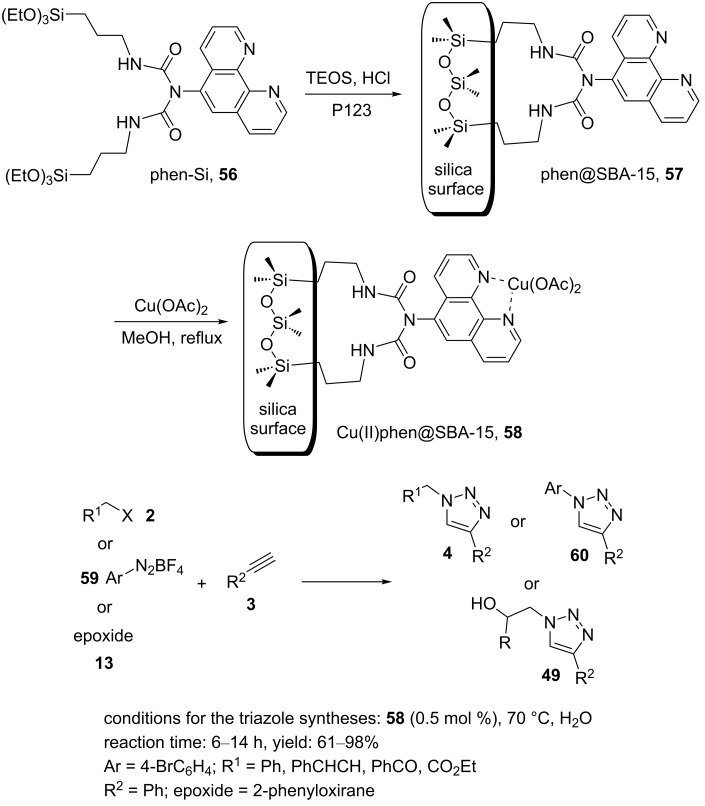
Synthetic route to the catalyst **58** and catalytic application of **58** in “click” reactions.

Further, Dufauda et al. reported a copper(II)–phenanthroline complex supported on the SBA-15 architecture, Cu(II)phen@SBA-15 (**58**) [31]. The preparation is outlined in Scheme 9: Initially, phen-functionalized mesoporous SBA-15 silica (phen@SBA-15, **57**) was prepared via dissolving pluronic P123 in acidic water at 35 °C. A mixture of phen-Si (**56**) and tetraethyl orthosilicate (TEOS) was added with stirring, followed by heating at 35 °C for 24 h. After transferring to a teflon bottle sealed in an autoclave and heating for 2 days at 100 °C, a solid was obtained, which was then filtered off, washed with distilled water, and dried. In the next step, the surfactant pluronic P123 was removed by the Soxhlet extraction method to produce a white solid. Then, **58** was synthesized using **57** and copper(II) acetate in methanol at reﬂux conditions for one day. The resulting powder was ﬁltered off, washed with methanol, and dried. Finally, excess copper was removed by the Soxhlet extraction method (Scheme 9).

The 1,3-dipolar reaction of organic alkynes, organic bromides (or aryldiazonium salts or epoxides), and sodium azide proceeded well using a catalytic amount of the synthesized catalyst **58** in water at 70 °C to create 1,4-disubstituted 1,2,3-triazole products in good to high yields (Scheme 9). In terms of recyclability, there was a continuous decrease in the catalytic activity of **58** from the first to the second to the third cycle of 98 to 55 to 26%. The deactivation of the catalyst was probably related to the remarkable decrease in pore volume and surface area that were proved by XPS surface analysis.

**Scheme 10:**
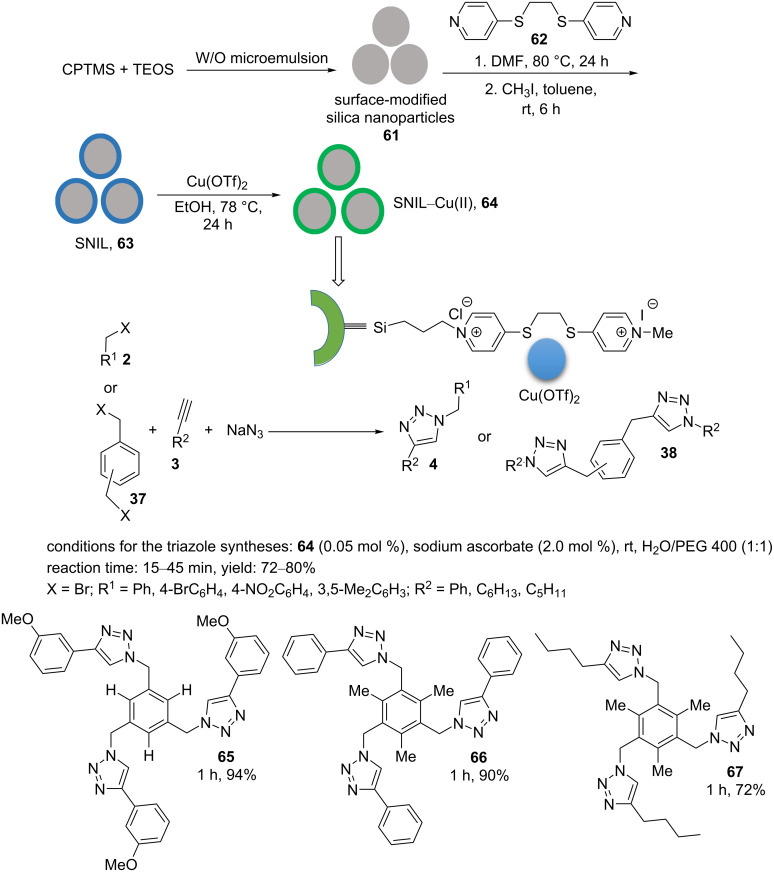
Synthetic route to the catalyst **64** and catalytic application of **64** in “click” reactions.

1,2-Bis(4-pyridylthio)ethane (**62**) was covalently grafted on the functionalized silica nanoparticles **61** by the reaction in DMF at 80 °C for 24 h. Through this, ionic liquid (IL)-supported silica nanoparticles were generated [32]. The resulting material was centrifuged, washed with methanol, and dried. Subsequently, the obtained nanoparticles and methyl iodide were reacted in dry toluene at room temperature. The resulting silica nanoparticles containing ionic liquid (SNIL, **63**) were centrifuged, washed with ether, and dried. The SNIL (**63**) were treated with Cu(OTf)_2_ in ethanol as a solvent at reflux for one day. Finally, this solid material was centrifuged, washed with methanol, and dried to produce the catalyst SNIL–Cu(II) (**64**) (Scheme 10) [32].

An interesting aspect of the heterogeneous catalyst SNIL–Cu(II) (**64**) is its successful application in the [3 + 2] cyclization synthesis of “click” products under mild reaction conditions. 0.05 mol % of **64** and 2 mol % of sodium ʟ-ascorbate were used to prepare a range of multitriazole products in polyethylene glycol as a green solvent at room temperature (Scheme 10). The authors proposed that sodium ʟ-ascorbate acted as a reducing agent in the reduction of Cu(II) to Cu(I). Aromatic/aliphatic terminal acetylenes and benzyl bromides bearing electron-rich and electron-deficient substitutions were efficiently transformed to the corresponding products in excellent yields. To show the high efficiency of this catalytic system, bis-“click” and tris-“click” reactions were also effectively performed (Scheme 10). The heterogeneous catalyst **64** could be used for six consecutive cycles without serious decline of the catalytic activity. The FTIR spectrum of the recovered catalyst **64** was almost the same as that of the freshly prepared catalyst. The high catalyst stability was confirmed by ICP analysis and a hot filtering test.

**Scheme 11:**
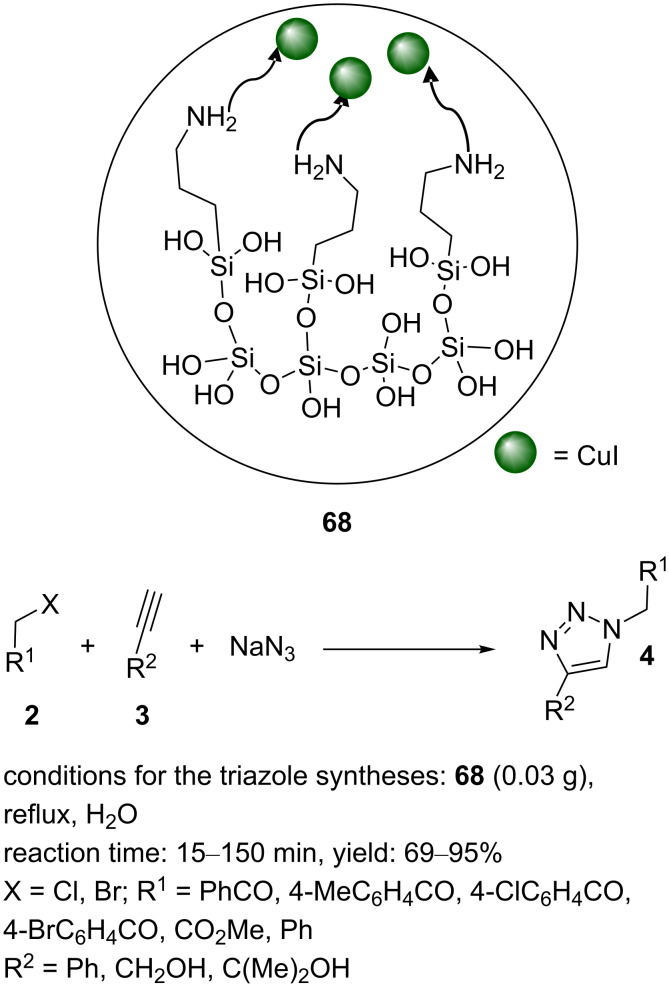
Chemical structure of the catalyst **68** and catalytic application of **68** in “click” reactions.

Heravi reported a procedure for the deposition of copper on (3-aminopropyl)triethoxysilane (APTES)/KIT-5 (CuI/AK, **68**) using CuI as the copper source [33]. The APTES/KIT-5, as a solid support, was added to a vessel containing CuI in acetonitrile. After stirring under reﬂux and a nitrogen atmosphere for 5 h, the solid product **68** was collected by filtration. The solid was washed with acetonitrile and dried before being used in “click” reactions. CuI/AK (**68**) furnished the desired products of the reaction of phenacyl halides (with Me, Br, and Cl substituents), benzyl halides or methyl iodide, and phenylacetylene or propargyl alcohol as substrates using a catalytic amount of this nanocatalyst in water as a green solvent under reﬂux. The triazoles were generated in short reaction times in good yields (Scheme 11). Finally, the heterogeneous catalyst **68** showed the benefits of good yields, short reaction times, high efficiency, not requiring chromatographic puriﬁcation, and high reusability.

In another method, sol–gel-entrapped copper in a silica matrix, the catalyst SiO_2_–Cu, was synthesized through a modiﬁed Stöber method [34]. NH_4_OH, water, and absolute ethanol were mixed and stirred. Afterwards, TEOS was slowly added to the mixture. After obtaining a white mixture, different amounts of CuI as the copper source were slowly added to the above mixture. Then, this was stirred at room temperature for 12 h. After centrifugation, the produced solid was washed with ethanol and water and then dried to create a ﬁne blue powder. In order to reduce the oxidation state of the copper component, the resulting powder was added to an aqueous solution of sodium ascorbate. Finally, the solid material was filtered off, washed with water and diethyl ether, and dried.

The catalyst was also used in the synthesis of 1,2,3-triazole products substituted at the 1- and 4-position through two-component or three-component “click” reactions. In the two-component reaction, acetylene compounds and organic azides were stirred in dimethylformamide or in a mixture of *t*-BuOH/H_2_O, 3:1, v/v as the solvent using diethylisopropylamine and SiO_2_–Cu. In the three-component example, acetylene species and organic halides as well as sodium azide were stirred in a mixture of *t*-BuOH/H_2_O, 3:1, v/v as the solvent in the presence of diethylisopropylamine and SiO_2_–Cu. In both cases, the SiO_2_–Cu catalyst was ﬁltered off and then, the triazole products were purified.

**Scheme 12:**
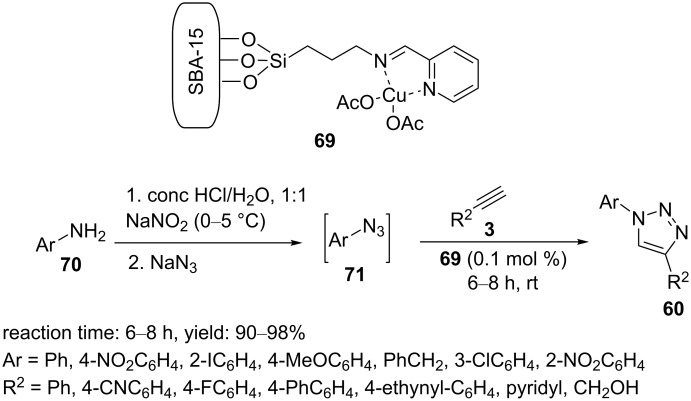
Chemical structure of the catalyst **69** and catalytic application of **69** in “click” reactions.

In another approach, 2-pyridine carboxaldehyde was reacted with 3-aminopropyl-functionalized SBA-15 in dry ethanol at 60 °C for one day to afford the SBA-15-supported imine ligand **69** (Scheme 12) [35]. The solid product was filtered off, washed with toluene/ethanol, and dried. The SBA-15-supported imine composite was added to an ethanolic solution of Cu(OAc)_2_, and the mixture was heated at reflux for 12 h. Finally, the material Cu@ImPy–SBA-15 (**69**) was collected, washed with ethanol, and dried. The catalyst was applied in the synthesis of triazole products via the reaction of in situ-prepared arylazides from anilines with aryl-/alkyl-alkynes in HCl/H_2_O using 0.1 mol % of Cu@PyIm–SBA-15 (**69**, Scheme 12).

### Copper anchored on functionalized carbon materials: efficient and recyclable catalysts for CuAAC reactions

Carbon nanomaterials have attracted the interest of researchers due to the high electrical and thermal conductivities, low production cost, oxidation stability, and low density, and diverse forms, such as graphene, fibers, horns, buds, onions, helices, etc. exist [36–55]. In addition, they are rather stable in strongly acidic and strongly basic solutions and capable to perform over a wide temperature range, increases the attractiveness of carbon nanomaterials [56]. Carbon materials have been used in different areas, including water purification, gas separation, fuel cells, photocatalysis, catalyst supports, etc. [57–62].

Different types of carbon nanomaterials, including a) zero-dimensional nanoparticles (NPs), b) one-dimensional nanotubes, c) two-dimensional graphene sheets, and d) three-dimensional mesoporous carbon have been reported in the literature [63].

In this regard, various functionalized carbon nanomaterials were employed in diverse organic reactions, including Suzuki reactions [64], Sonogashira reactions [65], transesterifications of triglycerides [66], hydrogenation reactions [67], N-heterocycle syntheses [68], cleavage of propargyl phenol ethers [69], etc. [70–75]. In this review article, we provide an overview on the synthesis and functionalization of carbon nanomaterials the catalytic application of these materials in CuAAC reactions.

**Scheme 13:**
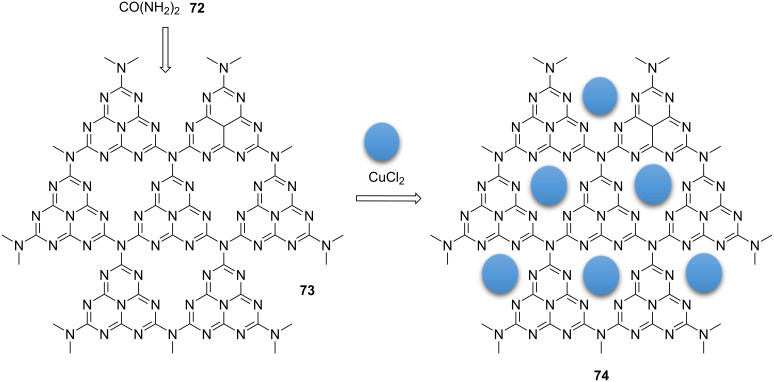
Synthetic route to, and chemical structure of the catalyst **74**.

Very recently, CuCl_2_ on fabricated graphitic polymeric C_3_N_4_-supported CuCl_2_ (Cu@g-C_3_N_4_, **74**) was prepared as an off-white bluish solid in two simple steps [76]. Generally, urea (**72**) was calcined and then, CuCl_2_ was immobilized using sonication (Scheme 13).

**Scheme 14:**
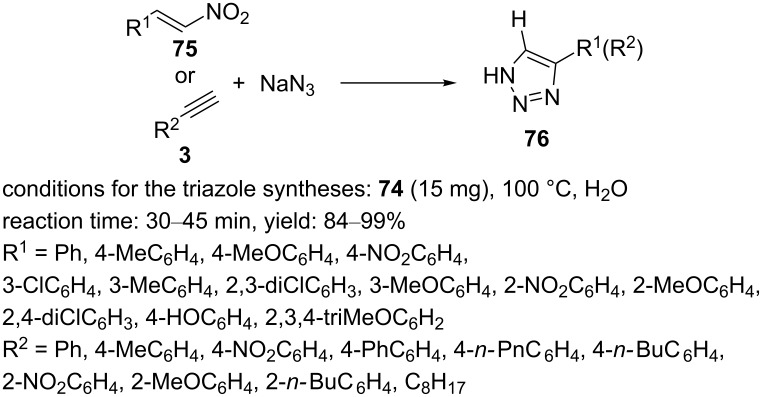
Application of the cayalyst **74** in “click” reactions.

The nanopolymeric catalyst was applied as a reusable nanocatalyst for promoting the 1,3-dipolar cycloaddition of nitroolefins/phenylacetylenes and sodium azide in water at ambient temperature. High yields of the 4-aryl-1,2,3-triazole **76** were obtained in short reaction times. The results indicated that ß-nitrostyrenes/phenylacetylenes containing electron-rich and -deficient groups could participate in the 1,3-dipolar cycloaddition reaction. Notably, the reaction was also extended to bistriazole derivatives (Scheme 14). The synthesized copper-catalyst **74** could be reused in up to ten consecutive cycles, and only very little leaching (0.08%) was observed.

**Scheme 15:**
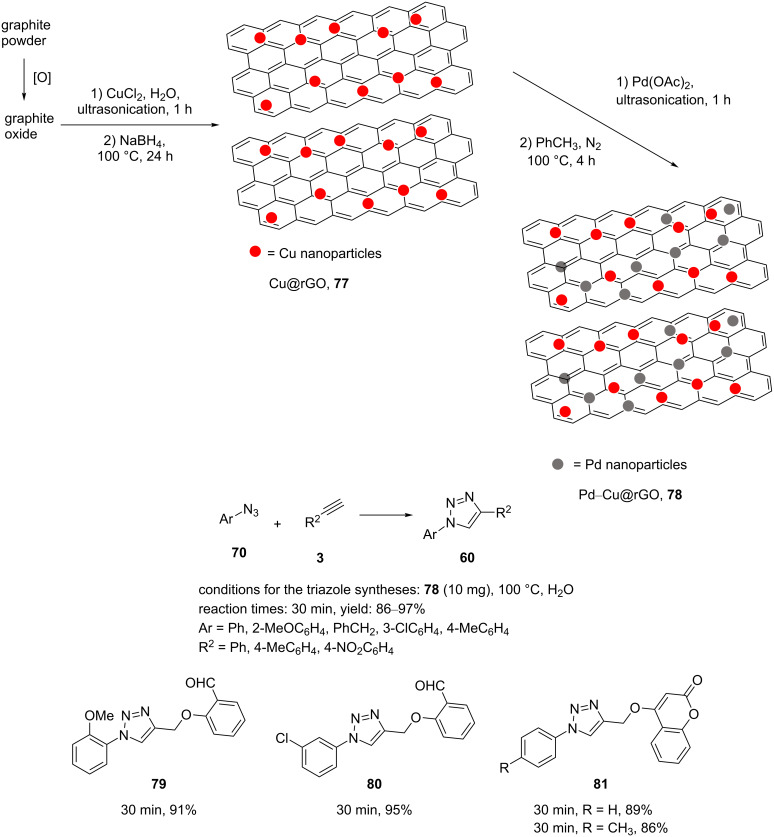
Synthetic route to, and chemical structure of the catalyst **78** and catalytic application of **78** in “click” reactions.

A novel and reusable, synergistic and dual catalyst Pd–Cu@rGO (**78**) was designed and synthesized by Naeimi and Ansarian through the decoration of reduced graphene oxide (rGO) with copper and palladium species (Scheme 15) [77].

In this study, graphite oxide (GO) was generated according to the modified Hummer’s method. Copper(II) was anchored on GO via ultrasonication. In the next step, copper ions were reduced by adding NaBH_4_. The mixture was then heated at 100 °C for one day, cooled, and filtered. The material Pd–Cu@rGO (**78**), as heterogeneous nanocatalyst, was prepared by anchoring Pd(OAc)_2_ on Cu@rGO in toluene via pyrolysis.

The catalyst was highly active in one-pot condensations of alkyl/aryl-substituted triazole heterocycles with cycloadditions and in “click” reactions of organic halides, terminal alkynes, and sodium azide. High yields of various triazole products were obtained when using 10 mg of the catalyst **78** in water at reflux for 30 min (Scheme 15). Moreover, this synergistic dual catalyst was also investigated in the stepwise generation of biaryl products with triazole. For this, 4-bromoaryl derivatives were initially treated with phenylboronic acid. The biaryl derivatives were then reacted with phenylacetylene to give triazole-containing biaryl compounds in high yields. Stilbenes were also applied in the reaction under similar conditions to obtain triazoles containing stilbenes. The nanocatalyst **78** could be readily recovered seven times without a significant loss of catalytic activity.

**Scheme 16:**
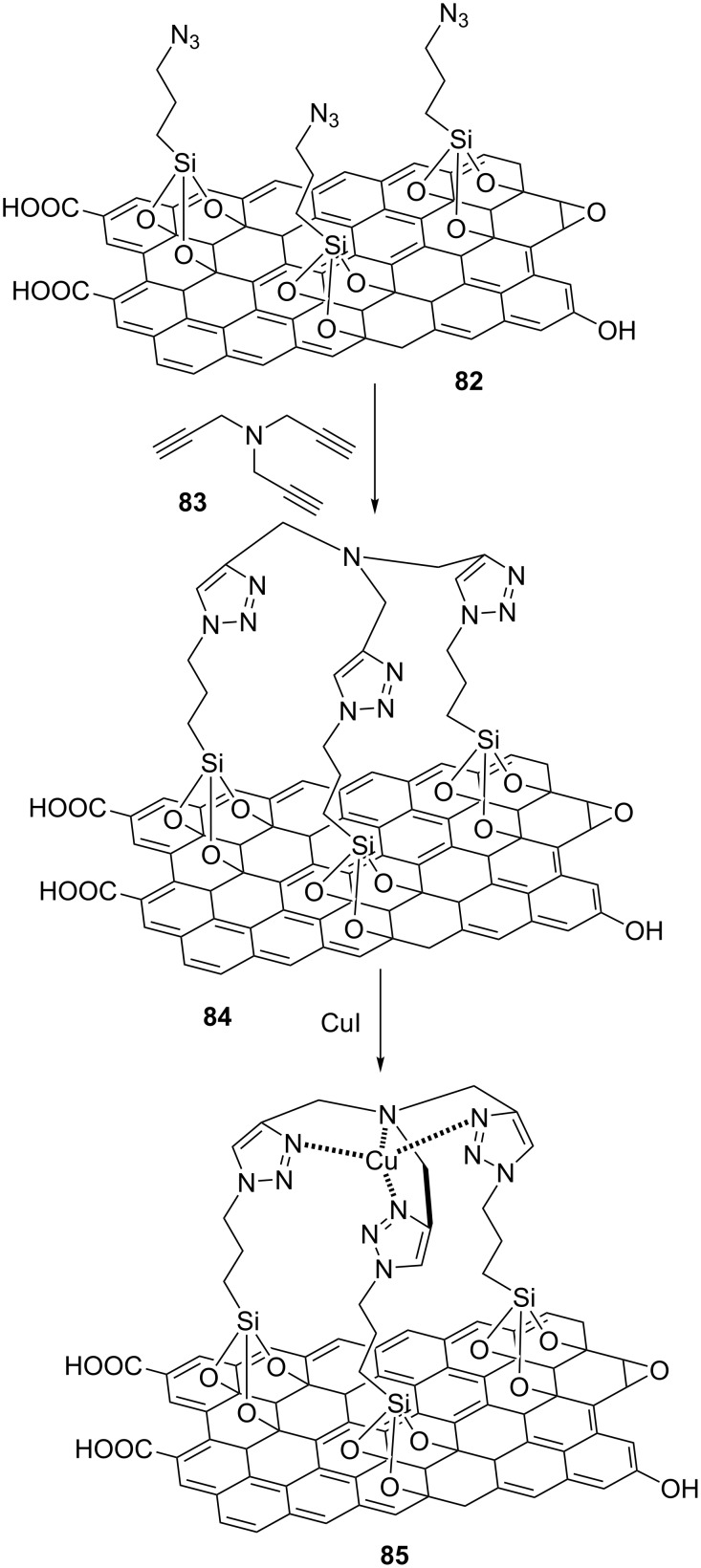
Synthetic route to the catalyst **85**.

Naeimi and Ansarian reported a new, effective, and reusable catalyst comprised of a polytriazole copper(I) complex supported on graphene oxide, GO@polytriazole–Cu (**85**), as outlined in Scheme 16 [78].

For this, 3-chloropropyltrimethoxysilane (CTS) was added to dispersed graphene oxide in dry toluene, and the mixture was refluxed for 2 days under a nitrogen atmosphere to afford chloro-functionalized GO (GO@CTS). The GO@CTS was sonicated in water for 5 min. NaN_3_ and KI were added to the reaction mixture, and this was heated at 60 °C for two days to afford the azido-functionalized GO GO@N_3_ (**82**). In the next time, tripropargylamine (**83**), CuSO_4_, and sodium ascorbate were added to dispersed **82**. The azide/alkyne “click” reaction proceeded well at rt over two days to produce the “click”-functionalized GO GO@PTA (**84**). CuI as a copper source was added to dispersed **84** in dry acetonitrile. The mixture was stirred at reflux for one day to give a copper complex supported by GO, GO@polytriazole–Cu (**85**, Scheme 16).

**Scheme 17:**
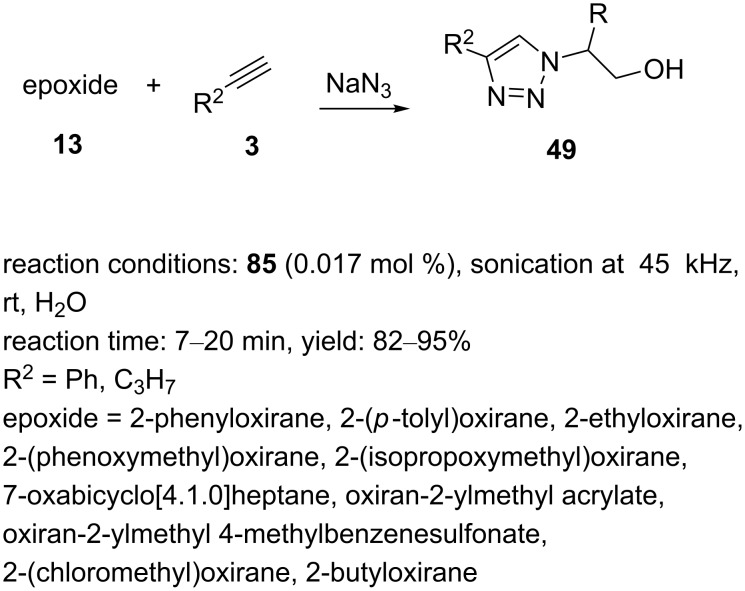
Application of the catalyst **85** in “click” reactions.

The obtained species **85** was then applied as a heterogeneous nanocatalyst in “click” reactions for the multicomponent synthesis of triazole products in water with ultrasound irradiation and 0.017 mol % catalyst loading. As such, a series of aryl/alkyl-substituted oxiranes was treated with sodium azide and nonactivated terminal alkynes, giving 1,2,3-triazole products substituted at the 1- and 4-position in good to high yields and with short reaction times (Scheme 17).

The nanocatalyst GO@polytriazole–Cu (**85**) could easily be recovered from the reaction mixture and was reused in five cycles, with no decline of its performance.

**Scheme 18:**
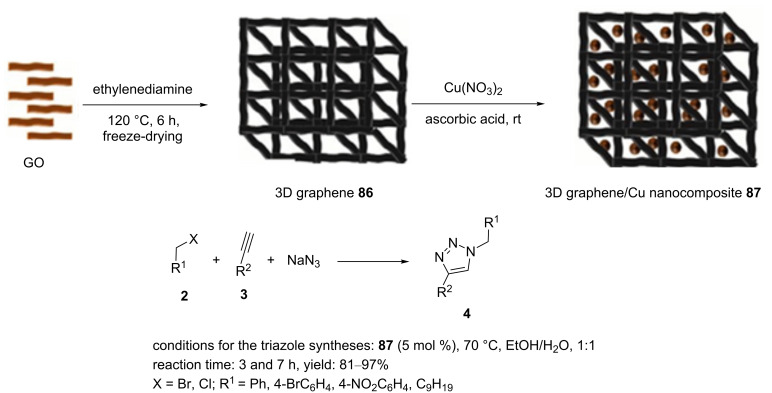
Synthetic route to the catalyst **87** and catalytic application of **87** in “click” reactions.

The design and synthesis of carbon nitride-supported copper nanoparticles was disclosed by Islam and co-workers (Scheme 18) [79–80]. The nanocatalyst **87** was successfully used as a high-performance photoreactor promoting “click” reactions of alkynes with organic azides under light irradiation in the absence of basic condition.

In the first step, photon energy caused an electron transition from the valence to the conduction band in gCN. In the next step, hot electron acted as a scavenger of the terminal proton of the alkyl-/arylacetylenes in order to facilitate the construction of the copper acetanilide species.

In the next example, copper nanoparticles-decorated three-dimensional graphene (3D graphene/Cu nanocomposite **87**) was reported as a catalyst to perform Huisgen 1,3‐dipolar cycloadditions. Therein, the GO material was mixed with EDA to produce a stable suspension that was transferred to a teflon‐lined autoclave. After heating at 120 °C for 6 h and then freeze-drying, a 3D graphene hydrogel was produced. After dissolving the 3D graphene **86** in water by ultrasonication, CuNO_3_⋅3H_2_O was added into the solution, and this was ultrasonicated for 3 h. ʟ-Ascorbic acid as a reducing agent was added into the solution and stirred for 24 h to produce a precipitate that was gathered and washed with water and ethanol (Scheme 18). It should be noted that 5.0 mol % of the 3D graphene/Cu nanocomposite **87** were sufficient to generate a high yield of the desired products in EtOH/H_2_O (1:1) at 70 °C. To screen the recyclability of **87**, the condensation of phenylacetylene and 1‐azido‐4‐nitrobenzene on a 4 mmol scale in EtOH/H_2_O (1:1) using 5 mol % of **87** was performed. After completion of the reaction, the heterogeneous catalyst was centrifuged and washed with ethyl acetate/water. This nanocatalyst could be reused in up to ten cycles.

A microwave-assisted method was developed for the preparation of 1,2,3-triazole products, substituted at the 1- and 4-positions, from benzyl halides, sodium azide, and nonactivated alkynes in a mixture of water and ethanol using a Cu(I) complex supported on graphene oxide, **88** [81], and the synthesis of “click” products by this new catalyst was successfully accomplished.

**Scheme 19:**
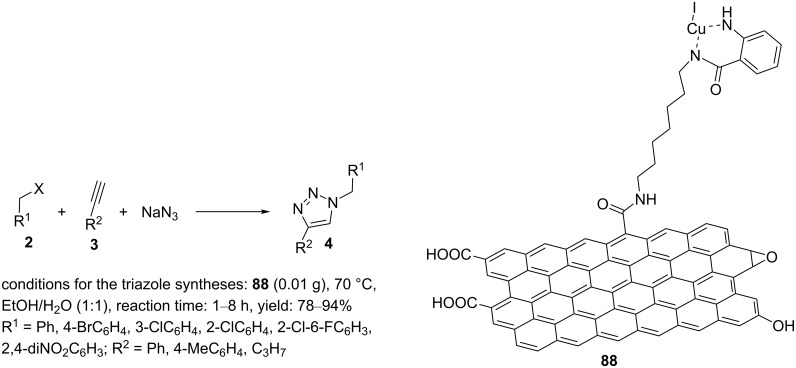
Chemical structure of the catalyst **88** and catalytic application of **88** in “click” reactions.

To synthesize the catalyst, ﬁrst, GO–COCl was created by the reaction of GO with thionyl chloride in DMF at reﬂux for 24 h. Then, the resulting GO–COCl compound was treated with 1,7-diaminoheptane to afford amino-modiﬁed graphene oxide (GO–CO–NH_2_), and this was subsequently reacted with isatoic anhydride (IA) in EtOH under reﬂux conditions for 24 h. GO–CO–NH–IA was then reacted with copper iodide under reﬂux conditions to obtain GO–NH–IA–Cu(I) (**88**, Scheme 19).

**Scheme 20:**
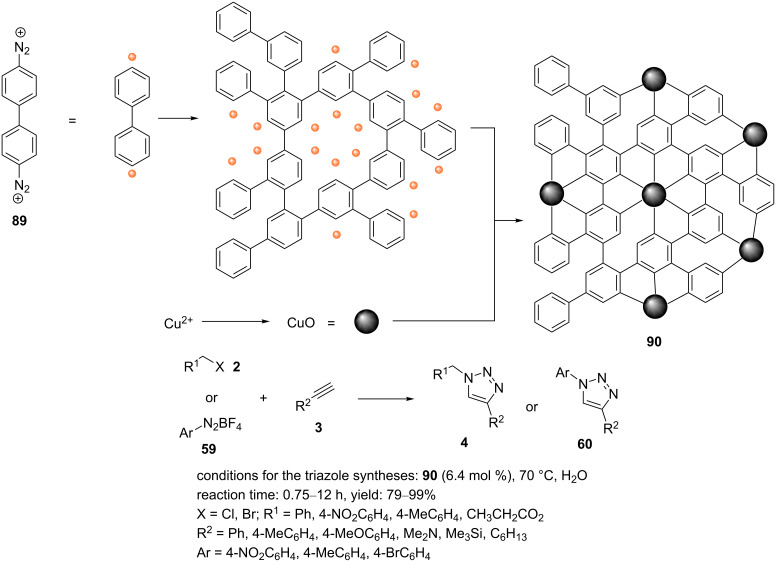
Synthetic route to the catalyst **90** and catalytic application of **90** in “click” reactions.

Gopidas et al. explored the catalytic activity of copper nanoparticles linked to organic frameworks (CNOF, **90**) for the preparation of 1,2,3-triazole derivatives substituted at the 1- and 4-positions under green reaction conditions [82]. The CNOF (**90**) worked well as catalyst for the Huisgen cycloaddition of benzyl azides and aromatic azides (generated from benzyl halides and diazonium salts, respectively) with aromatic and aliphatic alkynes (Scheme 20).

In the first step, 4,4'-biphenylbisdiazonium tetrafluoroborate (BPBDT, **89**) and CuCl_2_⋅2H_2_O were added to a water/toluene solvent system. Then, a methanolic solution of NaBH_4_ was added, and this was stirred for 6 h. The toluene layer was separated, washed with water, and subsequently diluted with methanol. The solid was filtered and washed with MeOH and was finally dried to afford the catalyst CNOF (**90**, Scheme 20). The nanocatalyst provided outstanding selectivity in the production of the 1,4-disubstituted 1,2,3-triazole products in an aqueous medium under aerobic conditions at 70 °C (Scheme 20).

In another example, GO was effectively modified with CuO nanoparticles through a facile pathway that can be seen below [83]. In the next step, GO was sonicated in isopropyl alcohol for 0.5 h to produce a black dispersion. After, copper acetate monohydrate was added to the GO to afford a brown dispersion that was stirred at 83 °C for 0.5 h. After the addition of water, the reaction mixture was stirred at 83 °C for 0.5 h and then cooled to ambient temperature. The solid product CuO–GO was centrifuged, washed with ethanol, and dried. It was demonstrated that the CuO nanoparticles–graphene hybrids could act as an effective catalyst for the “click” reaction in aqueous medium under aerobic conditions. The reusability of the catalyst CuO–GO was screened as well: the catalyst was recycled by simple filtration and washing with ethyl acetate, followed by water and acetone. The CuO–GO composite was reused five times without noticeable loss of catalytic performance.

**Scheme 21:**
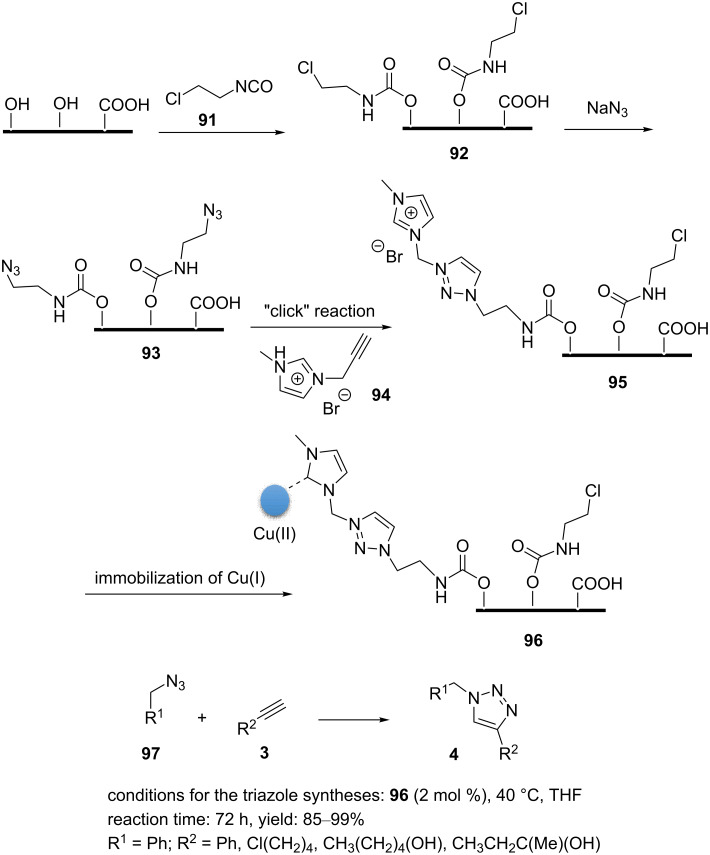
Synthetic route to the catalyst **96** and catalytic application of **96** in “click” reactions.

The carbon-supported copper nanomaterial **96** (Scheme 21) was prepared by trapping copper species on carbon graphene and carbon nanotubes [84]. This catalyst was found to be highly active in [3 + 2] cycloaddition reactions of halides, nonactivated terminal alkynes, and sodium azide (Scheme 21).

Copper(I) ions were attached to the surface of carbon graphene as outlined in Scheme 21. Therein, GO was generated by Hummer’s method, and ascorbic acid was used to produce chemically reduced graphene oxide (CRGO)–OH. The CRGO–OH and 2-chloroethyl isocyanate (**91**) were magnetically stirred in anhydrous DMF under nitrogen atmosphere to afford Cl–graphene (**92**). The azide-functionalized GO **93** was synthesized by the reaction of **92** with sodium azide in DMSO and heating for 48 h. The Cu(I)-catalyzed cyclization between CRGO–N_3_ (**93**) and 1-propargyl-3-methylimidazolium bromide (**94**) was used to attach the imidazolium moiety to the surface of the CRGO. Finally, a graphene-containing copper complex of N-heterocyclic carbene, (NHC)-Cu (**96**), was produced via proton exchange of the imidazolium scaffold with a Cu(I) (Scheme 21). The reaction between benzyl azide and alkynes proceeded in deuterated THF at 40 °C within 72 h using 2.0 mol % of the catalyst to obtain the desired products with good to high yields (Scheme 21). The nanocatalyst **96** could be readily recycled for use in more than 10 cycles.

**Scheme 22:**
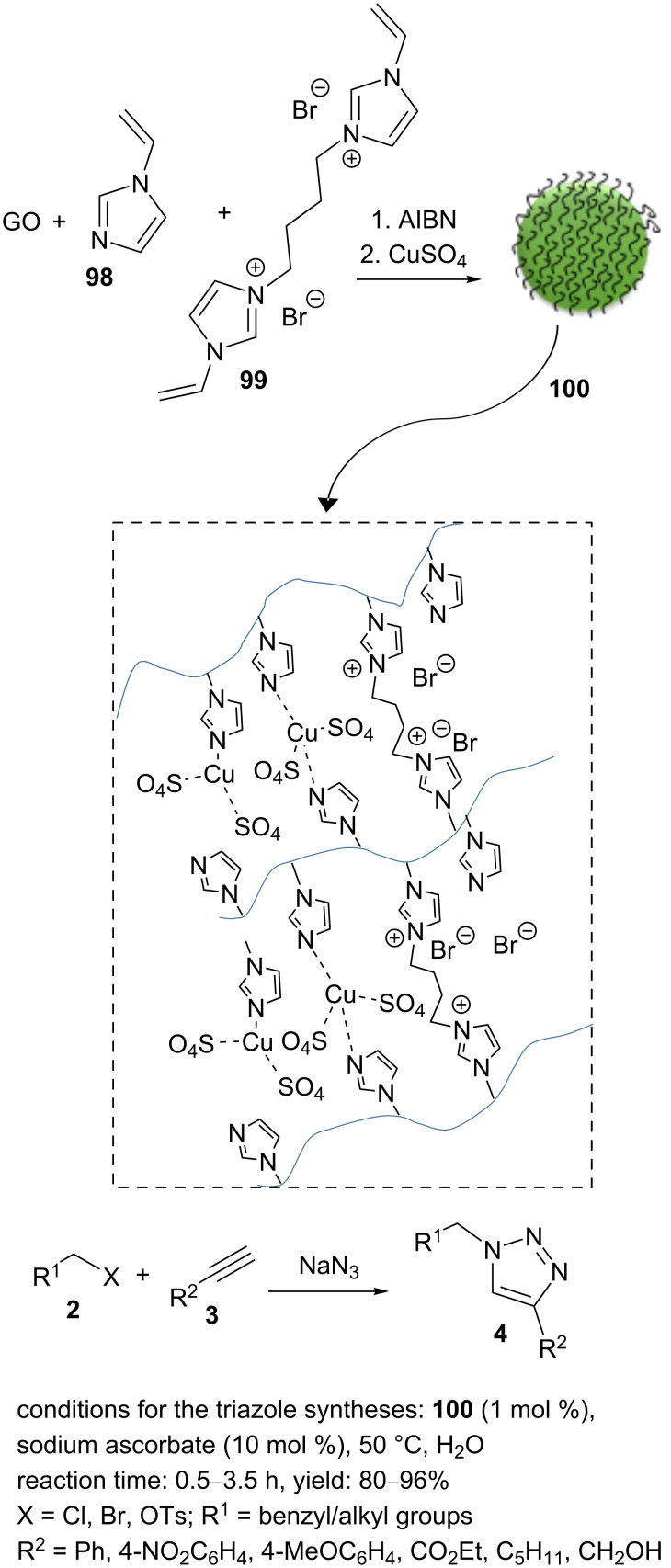
Synthetic route to the catalyst **100** and catalytic application of **100** in “click” reactions.

A mixture of GO, 1-vinylimidazole (**98**), and cross-linker **99** in ethanol was sonicated for 20 min [85]. Afterwards, the mixture was deoxygenated in an Ar atmosphere for 20 min. In the next step, polymerization was generated by adding AIBN (3 wt %). This was then heated at 70 °C for 24 h. This solid material was washed with MeOH and dried. The resulting GO/intrinsically microporous polymer (Pim) material and CuSO_4_ were added into water and heated at 50 °C overnight to afford the material GO/Pim/Cu (**100**, Scheme 22). The powder was collected by filtration and washed by water/methanol. The “click” reactions of alkyl halide, NaN_3_, and acetylene were performed successfully in the presence of **100** (1.0 mol %) as the catalyst and 10 mol % of sodium ascorbate as a reducing agent in water at 50 °C (Scheme 22). Further, the nanocatalyst **100** was durable and could be recycled eight times without any significant loss of catalytic activity [85].

**Scheme 23:**
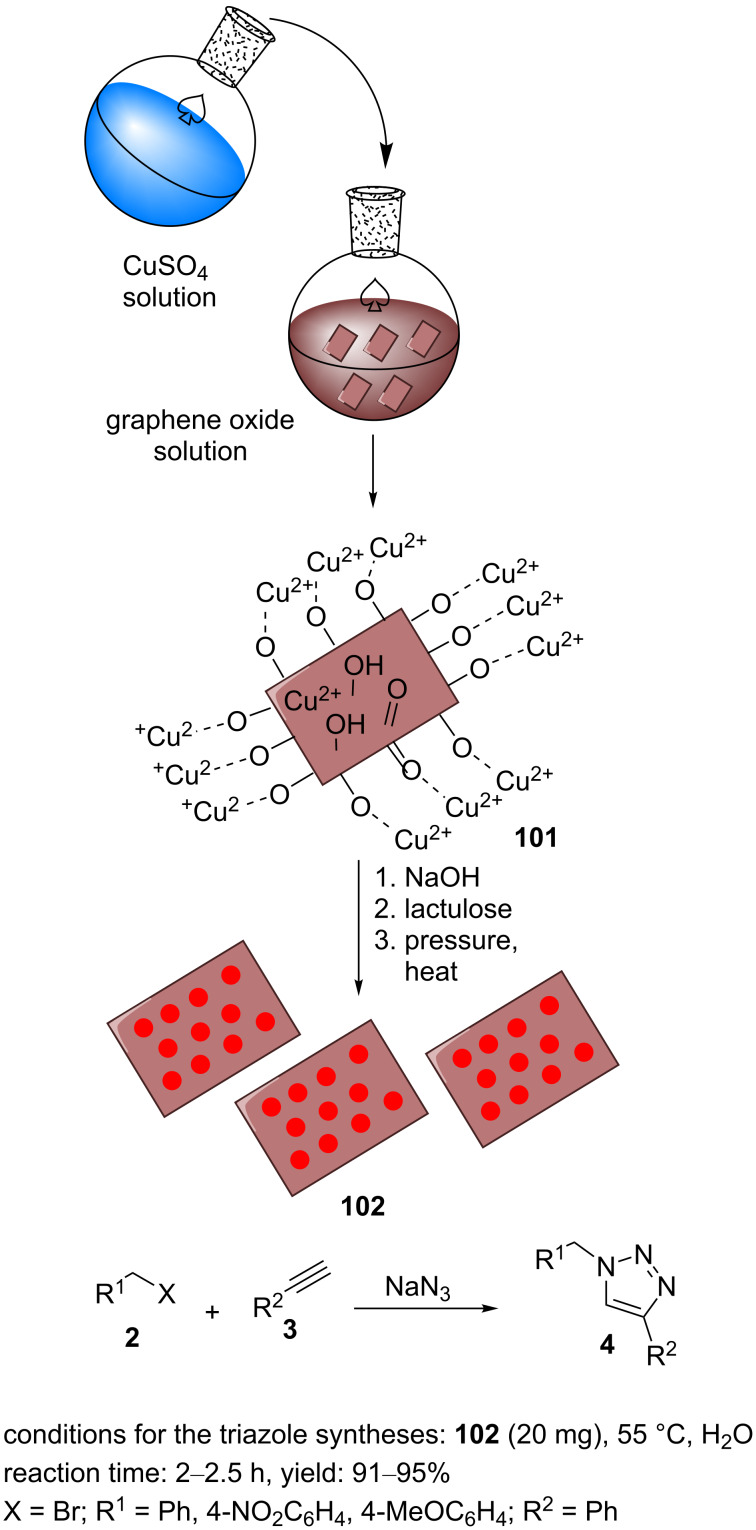
Synthetic route to the catalyst **102** and catalytic application of **23** in “click” reactions.

Chattopadhyay et al. prepared the nanocomposite rGO/Cu_2_O (**102**) via an in situ method [86]. Lactulose, a disaccharide, was used to reduce GO and the copper(II) source under alkaline conditions. Initially, the GO material was prepared from natural graphite powder via a modified Hummer procedure. Graphite powder, NaNO_3_, and concentrated sulfuric acid were mixed and vigorously stirred in an ice bath. After converting to a black slurry, KMnO_4_ was added and then, this was stirred for 4 h. The resulting mixture was diluted with water and stirred at room temperature for 2 h. The mixture was also diluted with hot water and then, H_2_O_2_ was added to adjust the pH value and to terminate the reaction. In the next step, the resulting mixture was washed with distilled water, and the pure graphene oxide powder was gathered by centrifugation and dried. The GO was dispersed in distilled water by ultrasonication. Subsequently, an aqueous solution of CuSO_4_ was added and stirred for 2 h. The pH value was increased to >10 by adding aqueous NaOH. An aqueous solution of lactulose was added as the reducing agent to the mixture to reduce GO and copper(II). After sonication for 10 minutes at 120 °C and 15 psi, rGO/Cu_2_O (**102**) was obtained. The nanoparticles of Cu_2_O with an average size of 5 nm were homogeneously dispersed throughout the rGO sheets (Scheme 23).

A mixture of phenylacetylene, benzyl bromides containing methoxy and nitro substitutions, and sodium azide was stirred using a catalytic amount of rGO/Cu_2_O (**102**) in water at 55 °C for 2 h to obtain 1,2,3-triazole derivatives substituted at the 1- and 4 position with high yields (Scheme 23). The catalyst **102** could be readily reused in six cycles [86].

In another study, charcoal-supported palladium and copper as a multitask nanocatalyst was used for one-pot Sonogashira-“click”/“click”-Heck sequences [87]. Palladium(II) acetate, copper(II) acetate, and charcoal were dispersed in methanol. In order to remove oxygen from the reaction mixture, hydrogen gas was passed through the medium. Then, the solution was stirred at ambient temperature for 12 h under a hydrogen atmosphere. Finally, the Pd–Cu/C catalyst was collected by filtration, washed with methanol, and dried.

An efficient one-pot sequential Sonogashira-“click” reaction toward heterocyclic structures was disclosed using the same catalyst. In this regard, trimethylsilylacetylene (TMSA) was selected for coupling with aryl iodides in the presence of a catalytic amount of Pd–Cu/C and PPh_3_ using Et_2_NH as a base in methanol at 120 °C under microwave irradiation for 20 min. In the next step, the azide derivative was added to the reaction mixture. Finally, the reaction mixture was irradiated at 120 °C for 10 min to the provide the triazole derivatives.

Binder et al. reported highly dispersed copper nanoparticles supported by graphene nanosheets [88]. After the preparation of GO by Hummer’s method, the GO was dispersed in water. In the next step, copper acetate was added under vigorous stirring, and this was reacted overnight to afford Cu(II)/GO. Finally, the reduction of the Cu(II)/GO composite was realized at 600 °C under an argon atmosphere to produce TRGO/Cu(I). According to STEM–EDXS, the quantities of copper and oxygen were in a ratio of 2:1, indicating the presence of Cu_2_O nanoparticles. The TRGO/Cu(I) catalyst (2 mol %) was applied in the synthesis of triazole derivatives by the reaction of benzyl azide with different alkynes at 40 °C for 48 h. In this protocol, the TRGO/Cu(I) species could be simply recovered and recycled four times without any significant loss of catalytic activity.

### Copper anchored on functionalized magnetic nanoparticles (MNPs): efficient and recyclable catalysts for CuAAC reactions

MNPs are of significant interest to chemists owing to a number of unique features, including their high structural diversity, large surface area, superparamagnetic behavior, ease of separation, and high safety. Indeed, materials including silica, carbon, etc. were combined with MNPs to produce new compositions that illustrated unique efficiency in the areas of catalysis [89], sensors [90], water purification [91], bioimaging [92], energy storage [92], drug delivery [93], and cancer detection [94].

In this regard, various functionalized MNPs were employed in diverse organic reactions, including Suzuki–Miyaura reactions, Sonogashira reactions, Ullmann coupling, hydrogenation reactions, oxidation reactions, hydroformylation reactions, and “click” reactions [89]. Because of the mentioned reasons, as well as due to the low toxicity and the magnetically reusability of these nanoparticles, they were also used in organic reactions. As such, in this review, we provide an overview on the synthesis and functionalization of MNPs and on investigations on their catalytic application in CuAAC reactions.

**Scheme 24:**
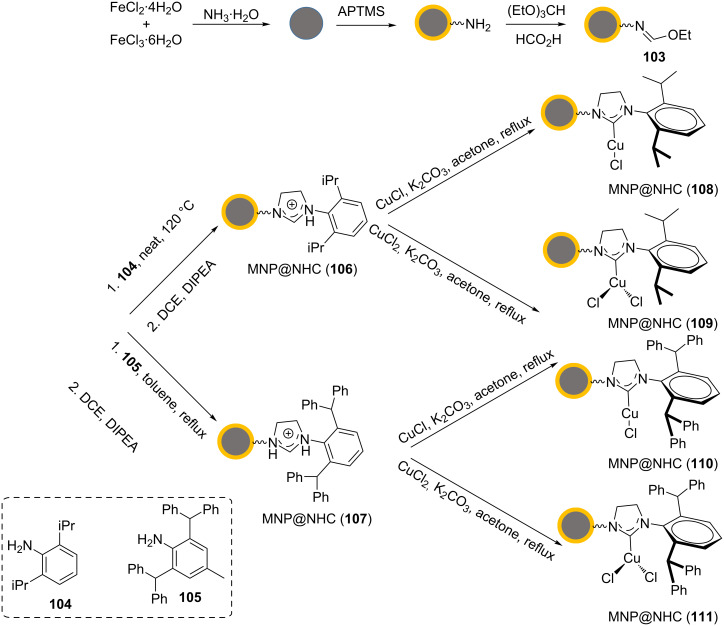
Synthetic route to the catalysts **108**–**111**.

Turkowicz, Wilczewska, and co-workers were the first to covalently immobilized a range of NHC–copper(I) and NHC–copper(II) complexes on MNPs [95]. The synthetic process for NHC–copper@MNPs **108**–**111** can be seen in Scheme 24. Initially, the magnetic nanoparticles were synthesized by heating a mixture of FeCl_2_⋅4H_2_O and FeCl_3_⋅6H_2_O in water at 80 °C in an ultrasonic bath. The pH value of the solution was increased to 11 by adding concentrated ammonia. In the next step, the MNPs were dispersed in EtOH by ultrasonication and diluted with EtOH. Then, the resulting mixture was reacted. After 15 min of stirring under argon, concentrated ammonia was added, and this was stirred for another 5 min. (3-Aminopropyl)trimethoxysilane (APTMS) was added dropwise to the mixture, and this was stirred at rt for 4 h. The silane-coated MNPs were magnetically separated. These nanoparticles were dispersed in triethylorthoformate and some drops of formic acid were added. The mixture was heated at reflux for 20 h to receive the new functionalized nanoparticles **103**.

The nanoparticles **103** were dispersed in 1,6-diisopropylaniline (**104**) by ultrasonication. The mixture was heated at 120 °C for about 20 h and then magnetically separated. After dispersing the resulting nanoparticles in 1,2-dichloroethane, *N*,*N*-diisopropylethylamine (DIPEA) was added. The mixture was heated under reflux conditions for 20 h to obtain the nanoparticles **106**, which were magnetically separated.

The nanoparticles **107** were also synthesized in multiple steps: The nanoparticles **103** were dispersed in toluene by ultrasonication. Then, 2,6-bis(diphenylmethyl)-4-methylaniline (**105**) was added to the mixture, and this was stirred at reflux over 20 h. The resulting material was magnetically separated. These nanoparticles were dispersed in 1,2-dichloroethane by ultrasonication. After adding *N*,*N*-diisopropylethylamine, the mixture was heated at reflux for 20 h. The nanoparticles **107** were magnetically separated and finally, the corresponding complexes of copper(I) and copper(II) with the NHC ligands, **108**–**111**, were prepared as shown in Scheme 24.

**Scheme 25:**
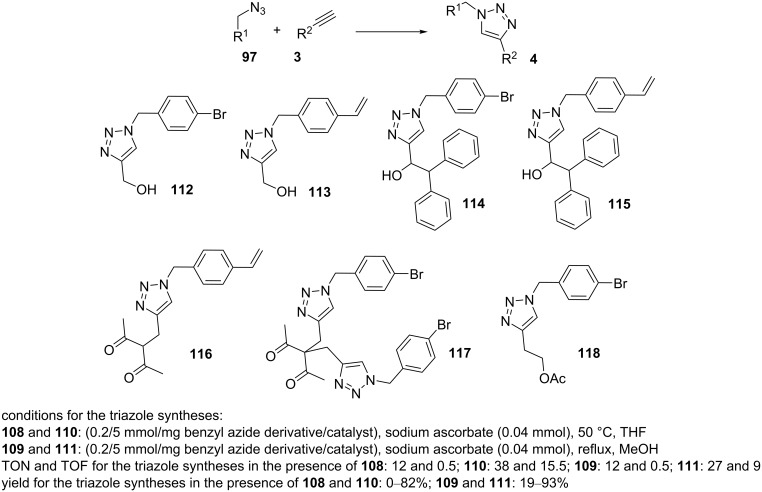
Catalytic application of **108–111** in “click” reactions.

The catalysts **108–111** were found to be effective in CuAAC reactions to form triazole products under mild conditions. The best results for NHC–copper(I) complexes were accomplished in THF at 50 °C. For the NHC–Cu(II) complexes **109** and **111**, the best yields were obtained in MeOH. Importantly, the NHC–Cu(II) complexes **108** and **110** required the addition of sodium ascorbate to reduce the oxidation state of Cu(II). In summary, the NHC–Cu(II) complexes **109** and **111** displayed higher catalytic activities than the NHC–Cu(I) complexes **108** and **110**. The catalyst **111** presented the best catalytic activity, high stability, and high selectivity (Scheme 25).

**Scheme 26:**
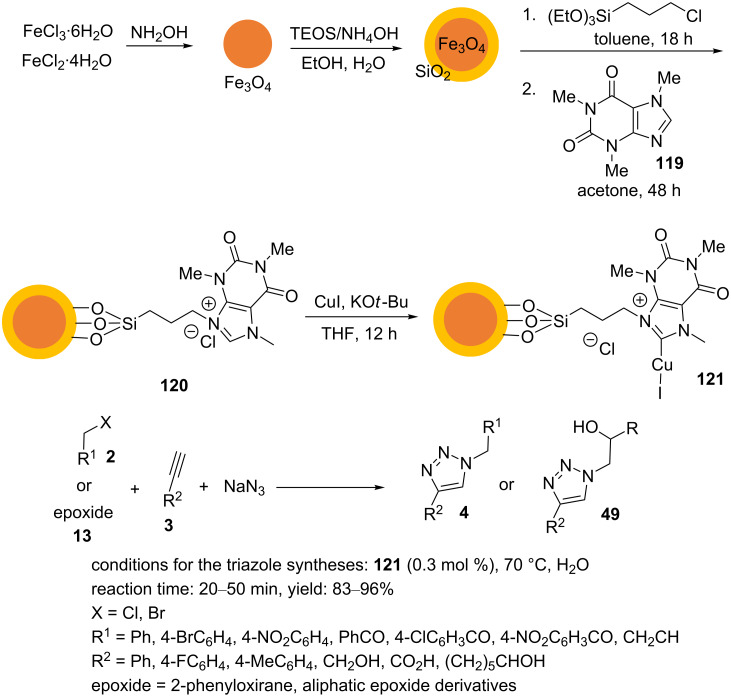
Synthetic route to the catalyst **121** and catalytic application of **121** in “click” reactions.

Recently, Heydari et al. reported a Cu(I)–caffeine complex supported on silica-coated magnetite nanoparticles, Fe_3_O_4_@SiO_2_–caffeine–Cu(I) (**121**), for “click” reactions in aqueous medium [96]. The heterogeneous catalyst **121** was prepared as outlined in Scheme 26. The salts FeCl_3_⋅6H_2_O and FeCl_2_⋅4H_2_O were dissolved in water, and the pH value was increased to 11 by adding an aqueous ammonia solution. The mixture was stirred at rt for 1 h and heated under reflux conditions for 1 h. The resulting magnetic nanoparticles were magnetically colleted from the aqueous solution and washed with water and ethanol. The Fe_3_O_4_ nanoparticles were dispersed in an ethanol/water mixture, and concentrated aqueous ammonia was added to the mixture to adjust the pH to approximately 10, and this was subsequently sonicated for 20 min. Then, TEOS was added to the mixture, and this was stirred at 40 °C for 12 h. The silica‑coated magnetite nanoparticles Fe_3_O_4_@SiO_2_ were magnetically separated from the aqueous solution and washed with water and ethanol. The Fe_3_O_4_@SiO_2_ nanoparticles were then reacted with (3-chloropropyl)triethoxysilane in toluene. After that, the mixture was heated at reflux for 18 h to obtain new nanoparticles. The resulting nanoparticles were magnetically collected and washed with toluene, methanol, and diethyl ether. These new nanoparticles were treated with caffeine (**119**) in dry acetone under reflux conditions for 48 h. The resulting nanoparticles **120** were magnetically collected and washed with methanol and acetone. Finally, the nanoparticles Fe_3_O_4_@SiO_2_–caffeine–Cu(I) (**121**) were prepared by the reaction of the nanoparticles Fe_3_O_4_@SiO_2_–caffeine (**120**), CuI, and KO*t*-Bu in dry THF at rt for 12 h. The final nanoparticles **121** were magnetically collected, washed with THF and dichloromethane, and dried.

Different organic halides (benzyl bromides, benzyl chloride, allyl bromide, aliphatic epoxides, and aromatic epoxides) were treated with sodium azide and phenylacetylene in the presence of a catalytic amount of the copper catalyst **121** in water at 70 °C (Scheme 26). These magnetically retrievable nanoparticles could be recycled five times.

**Scheme 27:**
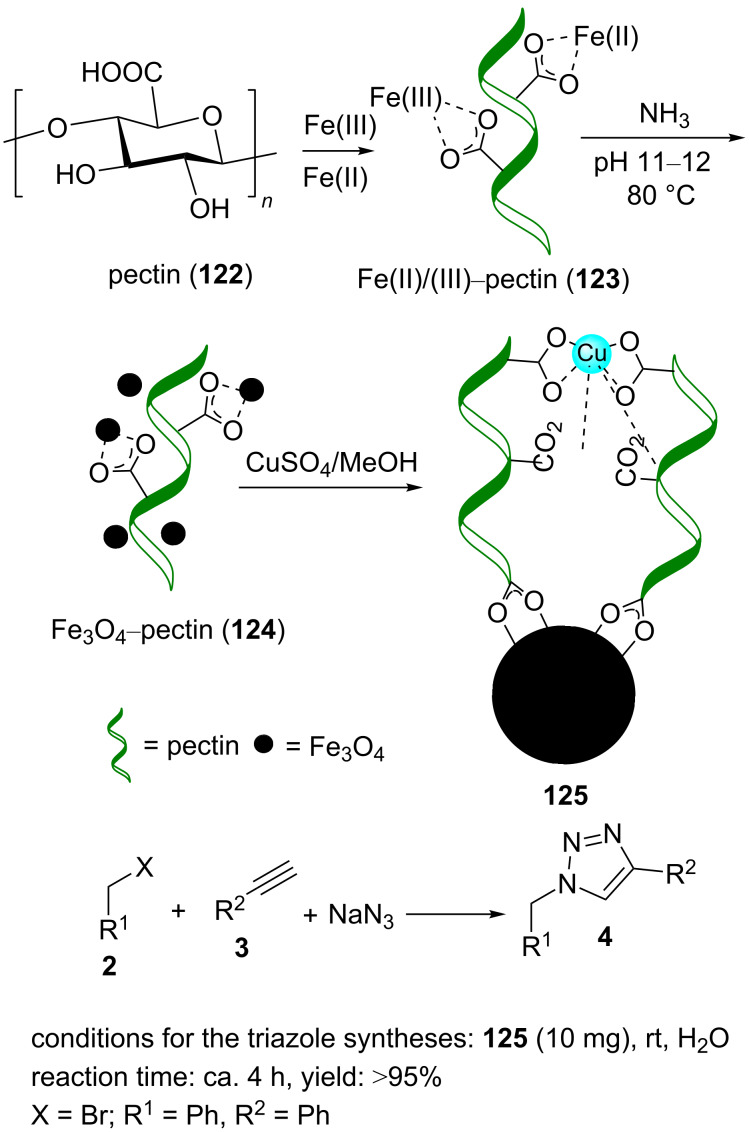
Synthetic route to **125** and application of **125** in “click” reactions.

Copper(II) was supported by carboxylic acid-rich Fe_3_O_4_–pectin (**124**) to aﬀord copper(II)-supported Fe_3_O_4_–pectin, Cu(II)@Fe_3_O_4_–pectin (**125**), as a superparamagnetic nanobiopolymer (Scheme 27) [97].

Briefly, **125** was synthesized by adding a methanolic solution of copper(II) sulfate to **124**, and this was allowed to stir for 2 h. The resulting nanoparticles were collected from the solution by centrifugation and washed/sonicated with methanol. Finally, **125** was dried before use in the reactions (Scheme 27).

Pectin (**122**) is a polymer that contains many carboxylate groups. These functional groups can chelate Fe_3_O_4_ nanoparticles (i.e., pectin includes the Fe_3_O_4_ nanoparticles as a shell). Generally, **122** is a branched macromolecule that is found broadly in nature. The promising aspects associated with pectin are biodegradability, nontoxicity, and inexpensiveness.

The catalytic application of Cu(II)@Fe_3_O_4_–pectin (**125**) was screened in the “click” reaction of phenylacetylene, benzyl bromide, and sodium azide to synthesize 1,4-disubstituted 1,2,3-triazole derivatives. Water was the best solvent for this reaction, and the temperature had a direct effect on the rate of the reaction. By increasing the reaction temperature to 70 °C, the reaction rate could be increased. The triazole product was generated at rt within 4 h (Scheme 27).

Cu(II)@Fe_3_O_4_–pectin (**125**) was reusable several times, and the results displayed good efﬁciency for up to 7 cycles. According to the TEM image obtained after the eigth cycle, the collapse of the catalyst was the reason for decreased activity of the catalyst at that stage.

**Scheme 28:**
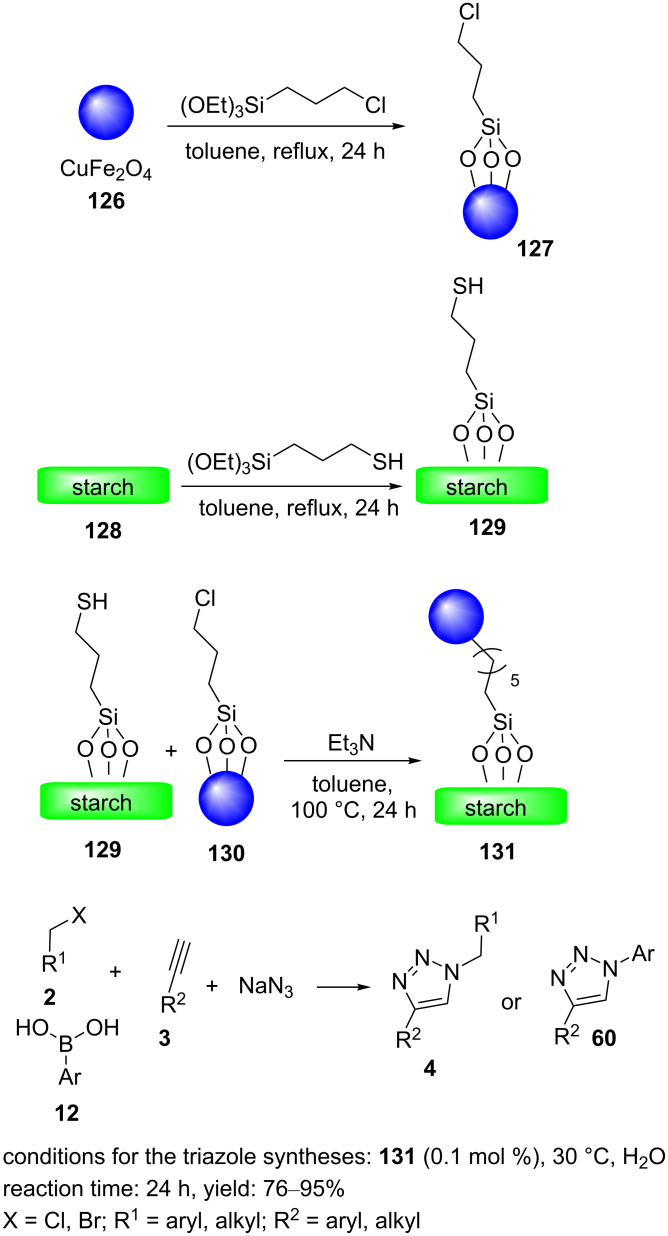
Synthetic route to the catalyst **131** and catalytic application of **131** in “click” reactions.

Gholinejad et al. reported the synthesis of copper ferrite nanoparticles-modified starch (CuFe_2_O_4_@starch, **131**) as a highly reusable nanomagnetic catalyst for “click” reactions and tandem syntheses of 1,2,3-triazoles substituted at the 1- and 4-position in water [98]. The synthetic approach to **131** is shown in Scheme 28.

First, magnetic CuFe_2_O_4_ nanoparticles **126** were treated with (3-chloropropyl)triethoxysilane in dry toluene to produce the magnetic nanoparticles Cl@CuFe_2_O_4_ (**127**). Subsequently, starch (**128**) was reacted with (3-mercaptopropyl)triethoxysilane in dry toluene to afford the starch-containing thiol-functionalized nanoparticles SH@starch (**129**). Next, **127** was grafted onto **129** in toluene at reflux conditions for 24 h to form the magnetic nanoparticles CuFe_2_O_4_@starch (**131**).

The catalytic activity of **131** was studied in the synthesis of 1,2,3-triazoles substituted at the 1- and 4-position by regioselective “click“ reactions of benzyl/alkyl bromides or arylboronic acids, sodium azide, and aromatic/alkyl alkynes in the presence of low copper catalyst **131** loadings (0.1 mol %) at 30 °C for 24 h (Scheme 28). The heterogeneous catalyst **131** could be recycled eleven times, with only a small decrease in catalytic activity. After that, the yield of the riazole product significantly decreased.

**Scheme 29:**
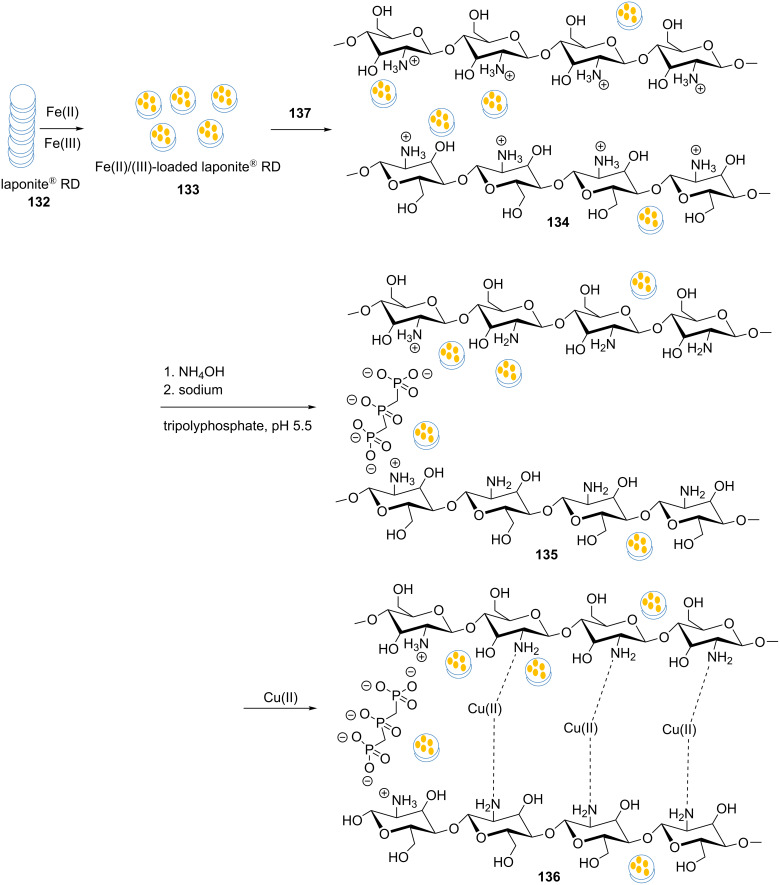
Synthetic route to the catalyst **136**.

A new magnetic catalyst **136** was prepared using a combination of chitosan (**137**) and laponite^®^ RD (**132**) [99]. The synthetic strategy to this catalyst is shown in Scheme 29. At ﬁrst, Fe(II)/Fe(III)-loaded laponite RD, **133**, was prepared by adding a solution of Fe(II)/Fe(III) ions to a dispersed solution of **132**. In the next step, a solution of **137** was added to the previous solution and then, the pH value was adjusted to 11, using ammonia solution, to achieve the magnetic nanocomposite. Sodium tripolyphosphate (**138**) was applied to create a crosslinked magnetic nanocomposite. It should be noted that **138** is a common crosslinker in the construction of **137**-based materials. Due to the incorporation of **137** and **132**, the capacity of the obtained magnetic nanocomposite **135** to complex Cu(II) ions was high. As such, copper ions were loaded into the magnetic nanocomposite, yielding **136**. The most important benefit of the magnetic nanoparticles being situated within the laponite^®^ discs was to prevent their aggregation.

**Scheme 30:**
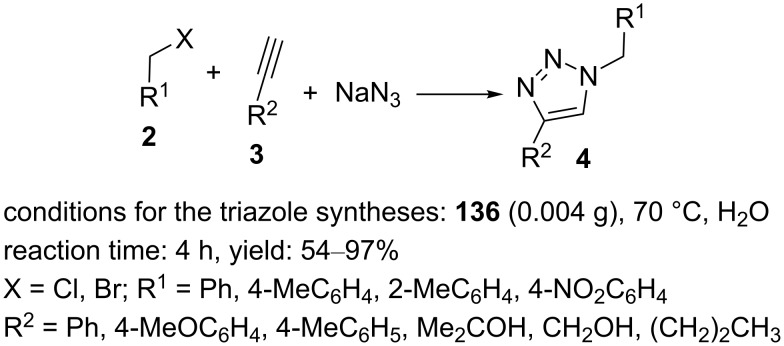
Application of the catalyst **136** in “click” reactions.

Moreover, the catalytic activity of the nanocomposite **136** was explored in 1,3-dipolar cycloadditions of alkynes with in situ-generated organic azides. The reactions proceeded well in an aqueous medium at 70 °C with 0.004 g of the catalyst (Scheme 30).

**Scheme 31:**
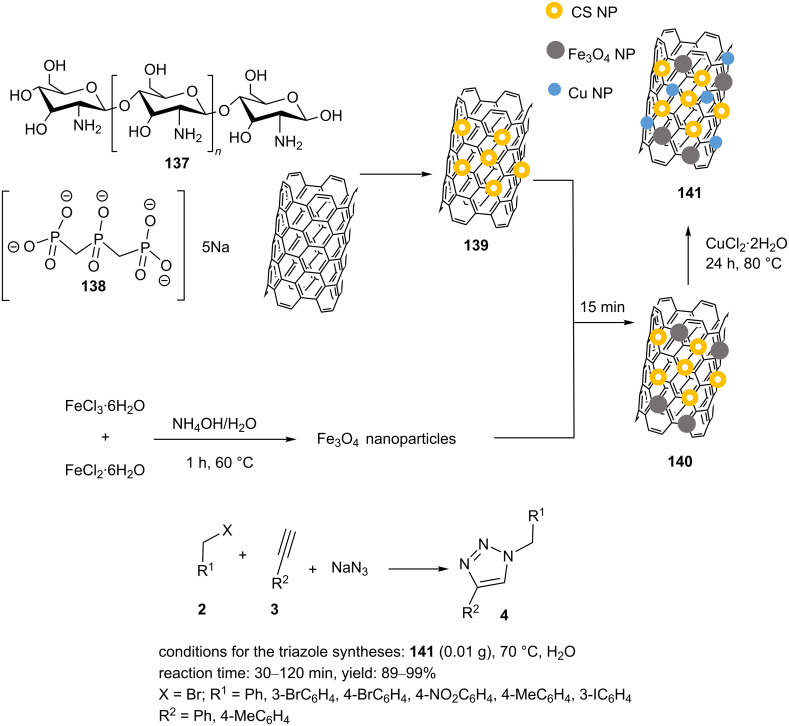
Synthetic route to the catalyst **141** and catalytic application of **141** in “click” reactions.

In another study, Shaabani reported crosslinked chitosan nanoparticles-anchored magnetic multiwalled carbon nanotubes Cu–CS NPs/MWCNT@Fe_3_O_4_ (**141**) as a bionanoreactor catalyst, and the catalytic activity of **141** in 1,2,3-triazole syntheses was explored [100]. A schematic pathway for the creation of **141** is presented in Scheme 31. In the first step, chitosan nanoparticles/multiwalled carbon nanotubes (CS NPs/MWCNT, **139**) were prepared by the addition of MWCNT–COOH to an acetic acid solution of chitosan (CS, **137**). Subsequently, a solution of sodium tripolyphosphate (**138**) was added to the previous solution. After stirring for 5 h, the mixture was sonicated at room temperature. The resulting nanomaterial was collected by centrifugation and rinsed with water. CS NPs/MWCNT (**139**) was then added to sonicated Fe_3_O_4_ nanoparticles in water. The mixture was sonicated for 15 min at room temperature, and **140** was separated, washed with deionized water/ethanol, and dried. The material **140** was then sonicated in water for dispersion. An aqueous solution of CuCl_2_⋅2H_2_O was added to the mixture and subsequently, ascorbic acid was applied. The resulting mixture was stirred at 80 °C for 24 h. Finally, Cu–CS NPs/MWCNT@Fe_3_O_4_ (**141**) was collected with a magnet and washed with deionized H_2_O/ethanol. Ultimately, the synthesized catalyst **141** was applied in the three-component synthesis of 1,2,3-triazole products in an aqueous solution at 70 °C (Scheme 31).

**Scheme 32:**
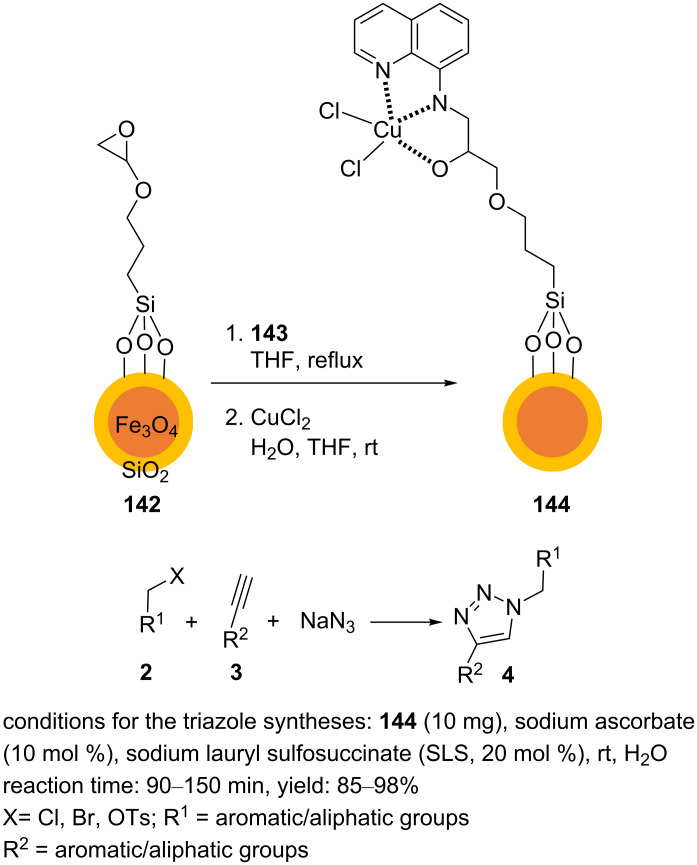
Synthetic route to the catalyst **144** and catalytic application of **144** in “click” reactions.

8-Aminoquinoline (**143**) was immobilized on Fe_3_O_4_@SiO_2_ functionalized with (3-glycidoxypropyl)trimethoxysilane (**142**) [101]. The supported quinoline ligand was used to coordinate copper ions. In this regard, (3-glycidoxypropyl)trimethoxysilane was added to a dispersed mixture of Fe_3_O_4_@SiO_2_ in dry toluene, and this was heated at reflux for 72 h. The resulting solid product was collected using a magnet. The obtained powdered material was washed with methanol and dried. The material **142** was dispersed in THF and then, **143** was added. The mixture was heated under reflux conditions for 24 h to afford aminoquinoline-functionalized ferrite (MNPs@FGly–8-AQ). The MNPs@FGly–8-AQ material was collected from the reaction mixture. An aqueous solution of Cu(II) was added to dispersed MNPs@FGly–8-AQ in THF, and this was stirred for 24 h. The synthesized material where Cu(II) ions were immobilized on 8-aminoquinoline-functionalized ferrite (MNPs@8–AQ⋅CuCl_2_, **144**) was collected using a magnet and washed with methanol (Scheme 32).

MNPs@8-AQ⋅CuCl_2_ (**144**) has been applied as a recyclable and eﬃcient nanocatalyst for the multicomponent one-pot synthesis of structurally diverse mono- and bistriazole products under green conditions. Reaction optimization result showed that a small amount of sodium ascorbate was needed to produce the desired triazoles. Although the reaction proceeded in the absence of an additive in water at 60 °C, the surfactant SLS allowed for a lower reaction temperature and an increase of the reaction yield (Scheme 32). The reusability of the catalyst **144** was screened over five cycles of the synthesis of 1-benzyl-4-phenyl-1*H*-1,2,3-triazole. Therein, the results indicated only a negligible decrease in the yield of the desired triazole.

**Scheme 33:**
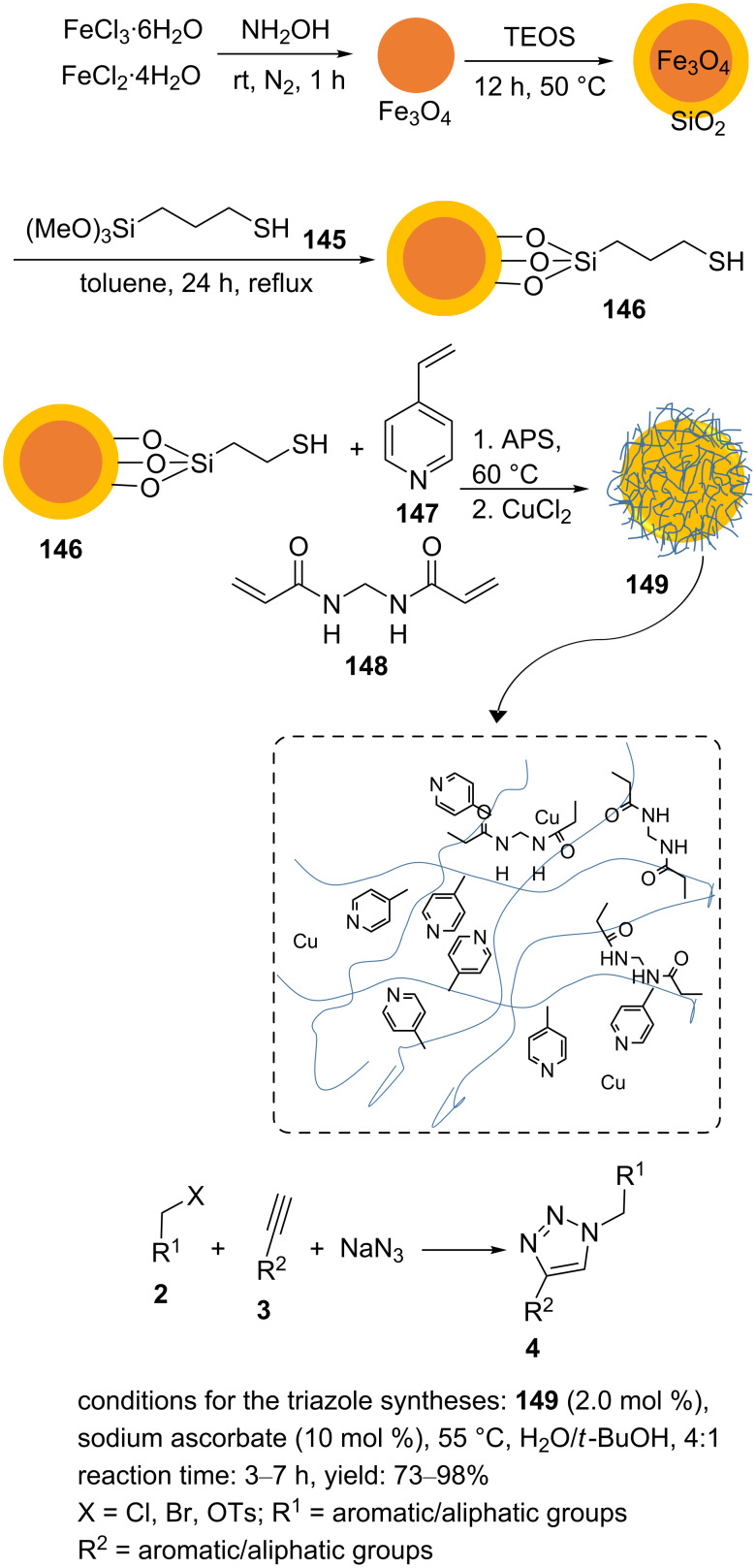
Synthetic route to the catalyst **149** and catalytic application of **149** in “click” reactions.

A magnetic heterogeneous copper catalyst **149** was designed, and the material was prepared by supporting copper ions by polymer-functionalized Fe_3_O_4_ (Scheme 33) [102]. Therein, the SiO_2_ shell was used to protect the Fe_3_O_4_ nanoparticles from agglomeration and oxidation. The surface of silica‐coated Fe_3_O_4_ nanoparticles (Fe_3_O_4_@SiO_2_ SMNP) was modified with (3‐mercaptopropyl)trimethoxysilane (**145**) to produce thiol‐modified silica‐coated magnetic nanoparticles (SMNP–SH, **146**). Then, **146** was dispersed in ethanol with ultrasonication. Next, 4‐vinylpyridine (**147**) and *N*,*N*′‐methylenebis(acrylamide) (MBA, **148**) were added to the mixture, and this was stirred for 10 min. Afterwards, ammonium persulfate (APS) was added, and the reaction mixture was heated at 60 °C. The resulting product was magnetically collected, washed with ethanol and water, and dried. Subsequently, the synthesized material was ultrasonically dispersed in THF, and CuCl_2_ was added. Because of the presence of nitrogen-containing ligands, the polymer-functionalized Fe_3_O_4_ could immobilize copper ions. Finally, the catalyst **149** was collected with a magnet and dried before being used in reactions (Scheme 33).

A range of 1,4‐disubstituted 1,2,3‐triazole derivatives was prepared using H_2_O/*t*‐BuOH as the solvent system at 55 °C and a catalytic amount of the catalyst **149** and alkyl halides, aryl/alkyl terminal alkynes, and sodium azide. The results showed that a small amount of sodium ascorbate was needed to reduce the oxidation state of the copper ions (Scheme 33). The catalyst **149** operated efficiently for nine circles in the Huisgen cycloaddition of phenylacetylene, benzyl bromide, and sodium azide without a considerable activity loss.

**Scheme 34:**
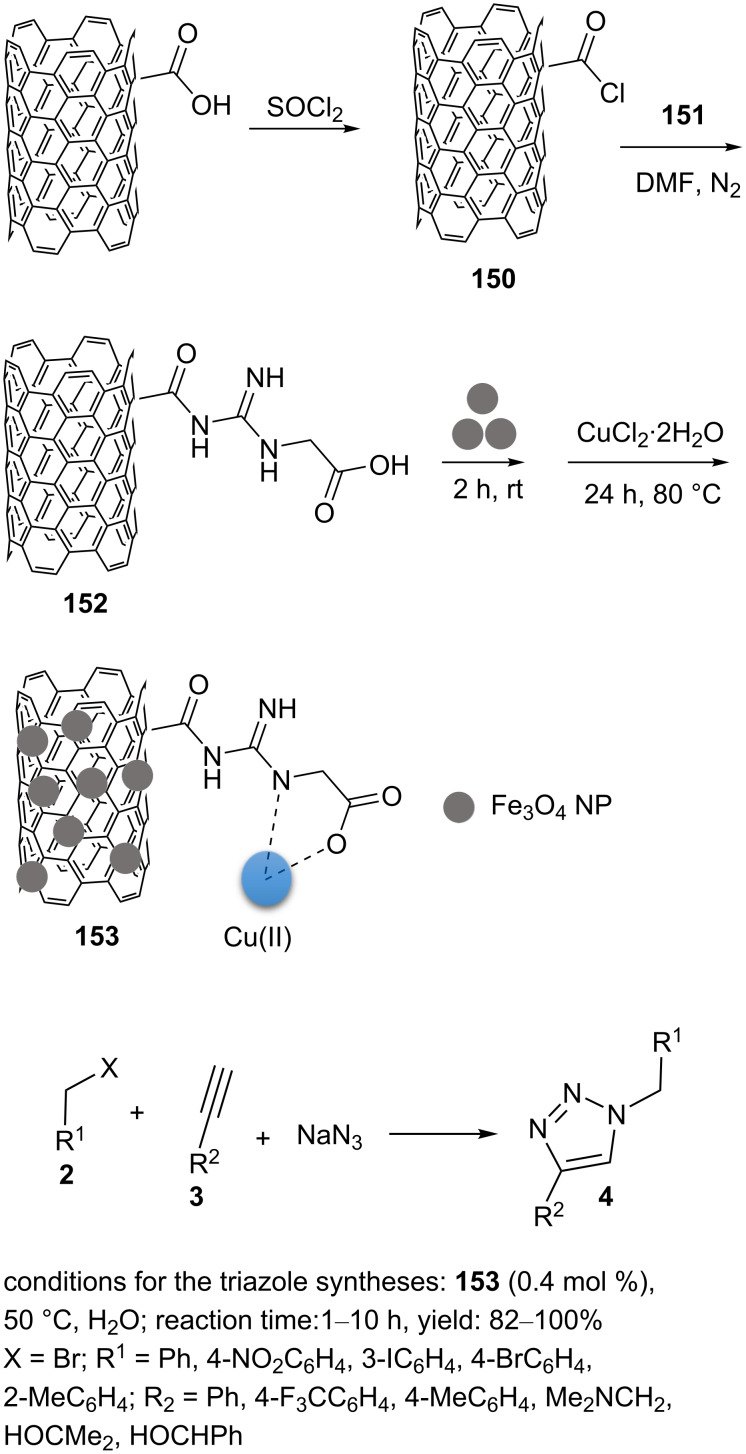
Synthetic route to the catalyst **153** and catalytic application of **153** in “click” reactions.

Shaabani and co-workers reported that copper supported on MWCNT–guanidine acetic acid@Fe_3_O_4_ (Cu/MWCNT–GAA@Fe_3_O_4_, **152**) could be used as a catalyst for the “click” synthesis of triazole products [103]. In the synthesis of **152** (Scheme 34), thionyl chloride was used to convert MWCNT–COOH into the corresponding acid chloride. The reaction was stirred at reflux conditions for two days at 75 °C. Excess SOCl_2_ was removed, and MWCNT–COCl (**150**) was washed with anhydrous THF. Then, guanidine acetic acid (GAA, **151**) was added to dispersed **150** in DMF, and this was stirred for 5 h at room temperature in an inert atmosphere. After evaporation of the solvent, the resulting solid was washed with acetone to yield MWCNT–GAA (**152**). A solution of CuCl_2_⋅2H_2_O in water was added dropwise to dispersed **152** in water. Then, ascorbic acid was added, and the reaction mixture was stirred at 80 °C for 24 h. After evaporation of the solvent, the precipitate was washed with deionized water and ethanol and dried to afford Cu/MWCNT–GAA (**153**). Species **153** was also prepared in situ: For this, **152** was added to dispersed Fe_3_O_4_ nanoparticles in water. The mixture was sonicated at room temperature for 2 h. Subsequently, an aqueous solution of CuCl_2_⋅2H_2_O was added to the mixture and then, ascorbic acid was added. After stirring at 80 °C for 24, Cu/MWCNT–GAA@Fe_3_O_4_ (**153**) was magnetically collected. The magnetic precipitate was washed with deionized water/ethanol and dried before being used in reactions (Scheme 34).

A low catalytic loading of **153** (0.4 mol %) was required to afford 1,2,3-triazoles in a one-pot three-component reaction of aromatic/aliphatic alkynes, alkyl halides, and sodium azide in water at 50 °C. The catalyst **153** was magnetically recovered from the mixture and recycled for subsequent reactions.

The magnetic core–shell material nanoFe_3_O_4_@TiO_2_/Cu_2_O (**155**) was designed and constructed according to the procedure listed in Scheme 34 [104]. An aqueous ammonia solution was added to dispersed Fe_3_O_4_ nanoparticles in a mixture of absolute ethanol and acetonitrile. The resulting mixture was stirred for 30 min, and an ethanol solution of tetrabutyl titanate (TBOT) was added dropwise to the above mixture under stirring at 30 °C. The stirring was continued for 1.5 h to yield the core–shell nanoparticles Fe_3_O_4_@TiO_2_ (**154**). The solid product was magnetically collected and washed with absolute ethanol. Following this, an aqueous solution of CuCl_2_ was added to **154** dispersed in water and then, an aqueous solution of NaOH was added dropwise under ultrasonication to obtain Cu(OH)_2_⋅NH_2_OH⋅HCl, which was immediately added to the previous solution over 5 s. After 1 h, the mixture was centrifuged. Finally, the top layer was decanted and the solid product was washed using water/ethanol to afford brown nanoFe_3_O_4_@TiO_2_/Cu_2_O (**155**).

**Scheme 35:**
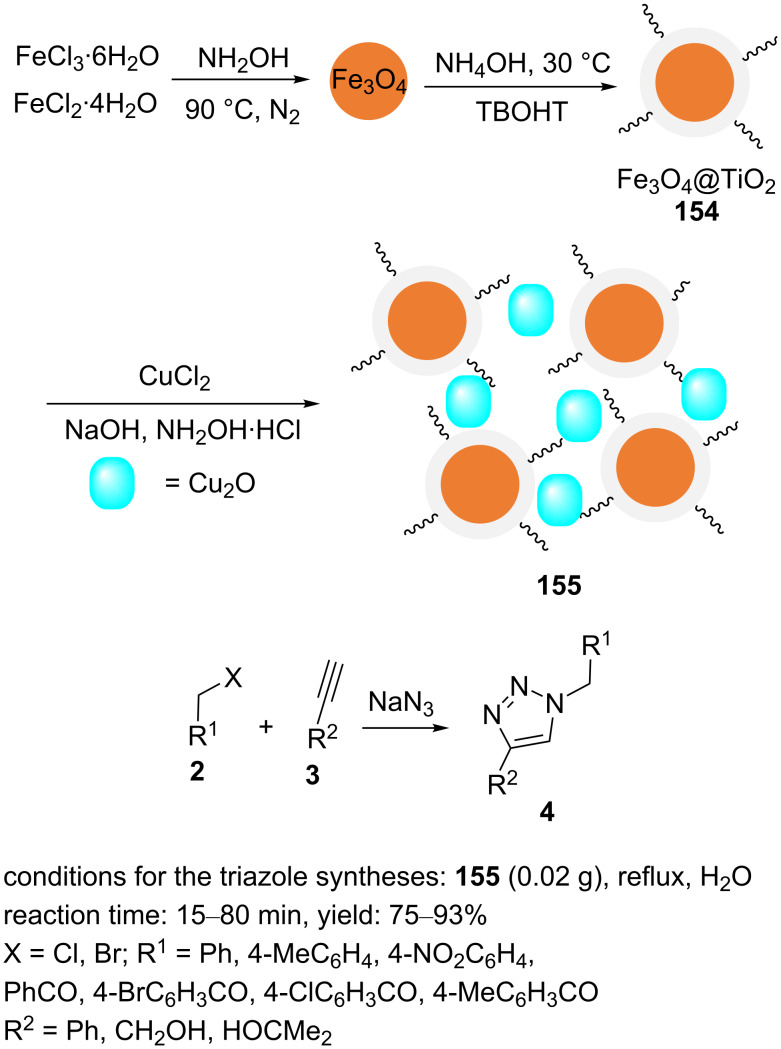
Synthetic route to the catalyst **155** and catalytic application of **155** in “click” reactions.

The core–shell composite **155** was used as a nanomagnetic catalyst for the regioselective synthesis of 1,2,3-triazole products. A series of 1,4-disubstituted 1,2,3-triazoles was obtained through the one-pot three-component reaction of nonactivated aryl/alkyl alkynes, alkyl halides, and sodium azide using 0.02 g of the catalyst in H_2_O under reflux conditions (Scheme 35).

The nanomagnetic catalyst **155** could be kept in a tightly closed vessel for several months without any decrease in activity, without any color change, and was reused four times without suffering from activity loss.

**Scheme 36:**
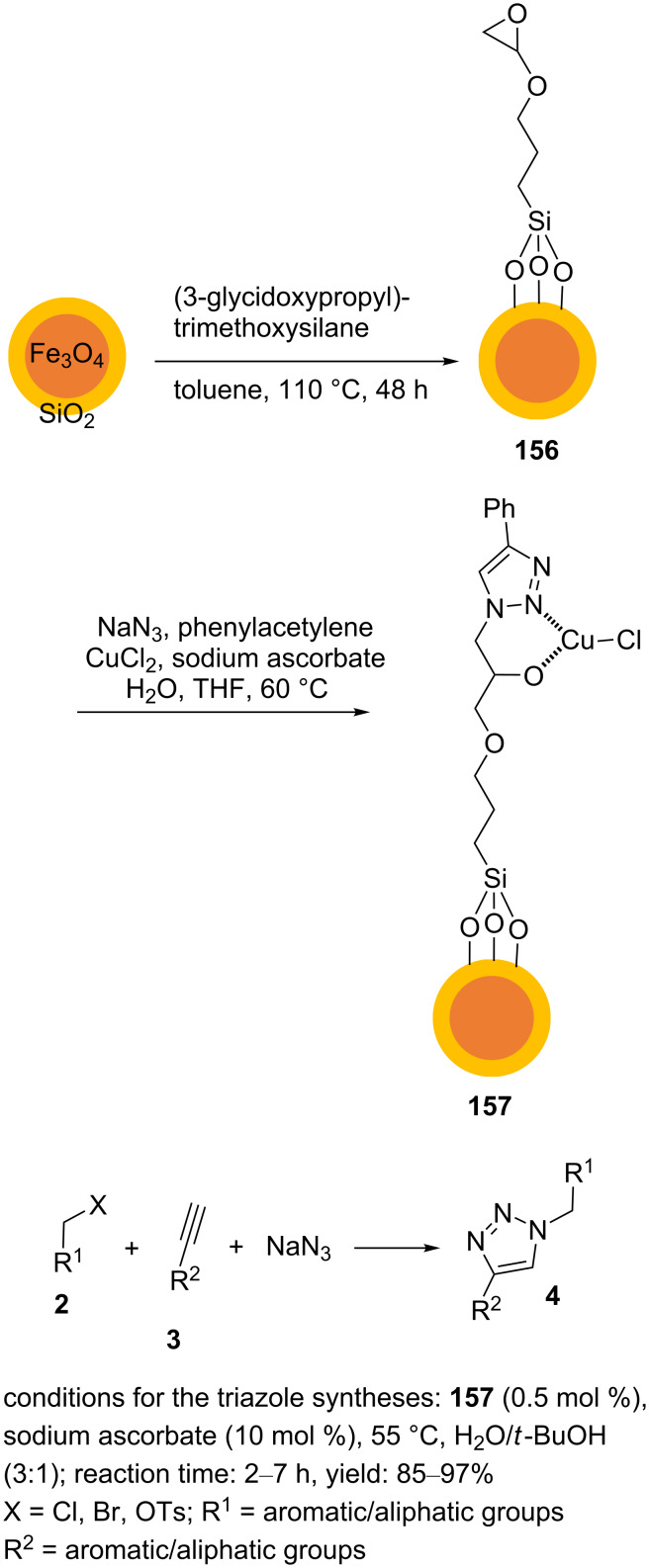
Synthetic route to the catalyst **157** and catalytic application of **157** in “click” reactions.

Moghaddam et al. immobilized a Cu–1,2,3-triazole complex on magnetic nanoparticles coated with silica (**157**) as a nanostructured heterogeneous catalyst [105]. The catalyst **157** was constructed in multiple steps, as shown in Scheme 36. First, 3-glycidoxypropyltrimethoxysilane on Fe_3_O_4_@SiO_2_, **156**, was formed using the reaction between Fe_3_O_4_@SiO_2_ magnetic nanoparticles and 3-glycidoxypropyltrimethoxysilane in refluxing toluene for 48 h. The solid product was then washed with methanol and dried. In the next step, these modified magnetic nanoparticles and phenylacetylene were added to a mixture of copper(II) chloride, sodium ascorbate, and sodium azide dissolved in a THF/water mixture. This was heated at 60 °C for 10 h while stirring. The resulting nanomaterial **157** was collected using an external magnet, washed with methanol, and dried. Aryl-/alkylacetylenes, alkyl halides, and sodium azide, the catalyst, and monosodium ascorbate as well as a mixture of H_2_O and *t*-BuOH as the solvent were added to a glass tube and heated at 55 °C for 4 h while stirring (Scheme 36).

In the synthetic route outlined above, no column chromatography was required, and the “click” products were purified by recrystallization from ethyl acetate/*n*-hexane. A magnet was applied to recover the catalyst **157** from the reaction mixture, and the catalyst showed good reusability in these Huisgen cycloaddition reactions.

1,4-Disubstituted ß-hydroxy-1,2,3-triazoles were synthesized via a novel one-pot reaction between aliphatic/aromatic epoxides, aliphatic/aromatic alkynes, and NaN_3_ in water at 60 °C using magnetic CuFe_2_O_4_ nanoparticles [106]. This procedure involved a reusable magnetic nanocatalyst, aqueous medium, no additives, and no requirement for the handling of organic azides as these were formed in situ during the reactions.

A nanomagnetic biocatalyst was prepared using isinglass (IG) as an attractive biopolymer [107]. IG consists predominantly of the protein collagen. The presence of various residues, including leucine, tyrosine, threonine, alanine, valine, hydroxyproline, proline, serine, arginine, aspartic acid, histidine, glycine, glutamic acid, phenylalanine, hydroxylysine, methionine, isoleucine, and lysine on the surface of IG renders it a natural, low-cost, and eco-friendly material for the coordination of metal ions.

IG was washed, dried, and ground in a ball mill machine. The obtained powder and Fe_3_O_4_ nanoparticles were treated using glutaraldehyde (GA) to form the magnetic isinglass nanoparticles IG@Fe_3_O_4_. Then, IG@Fe_3_O_4_ and Cu(OAc)_2_ were reacted in water at rt for 8 h. Finally, the Cu–IG@Fe_3_O_4_ nanocatalyst was washed with water, ethanol, and acetone and dried. The synthesized nanocatalyst was applied for the “click” synthesis of triazoles from organic halides, organic alkynes, and sodium azide using K_2_CO_3_ in water at 70 °C. The reusability of the magnetic material Cu–IG@Fe_3_O_4_ was analyzed, confirming good reusability. The Cu–IG@Fe_3_O_4_ nanocatalyst could be separated from the reaction mixture using a magnet and was recycled for use in five reactions.

**Scheme 37:**
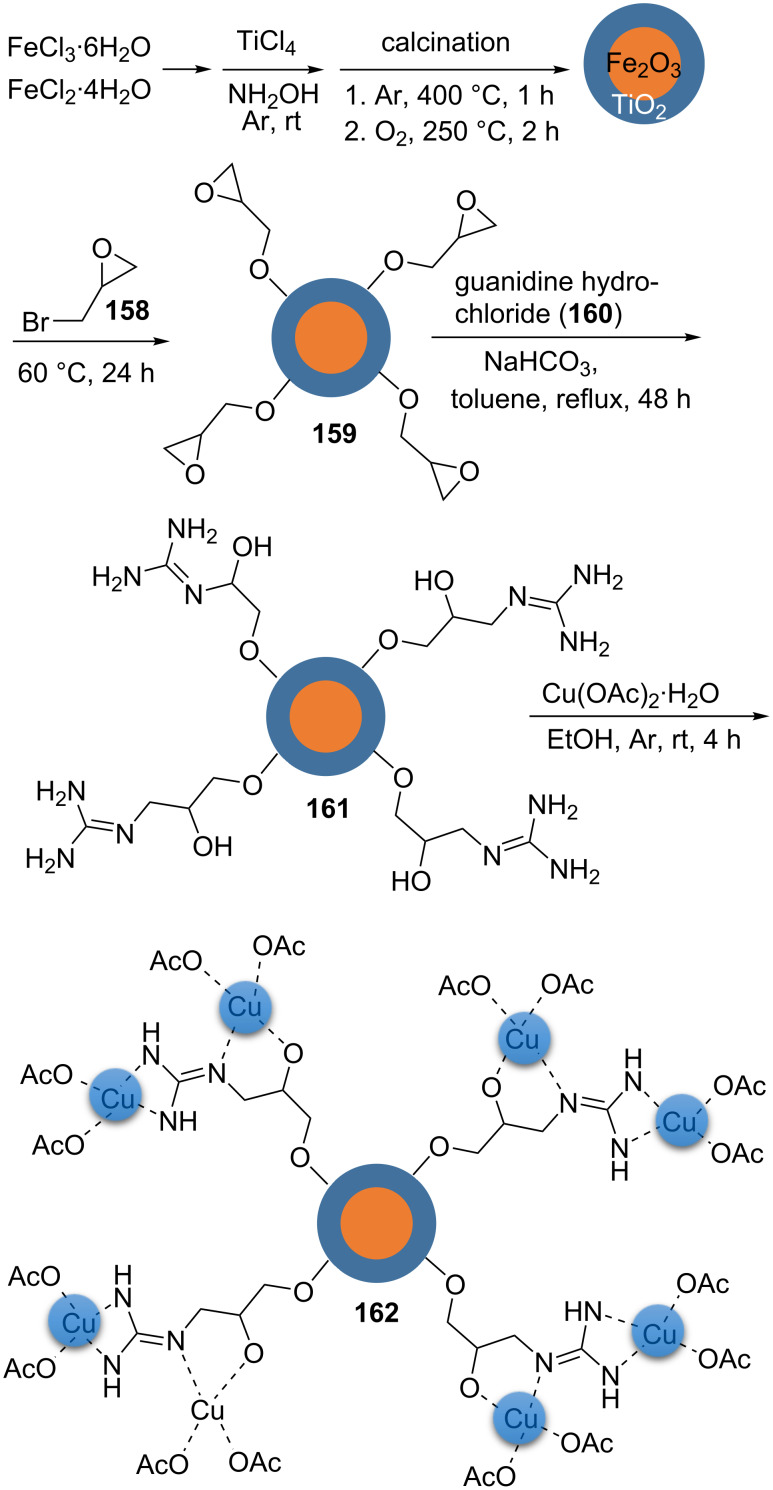
Synthetic route to the catalyst **162**.

A practical, efﬁcient, and simple strategy for the immobilization of Cu(II) on functionalized γ-Fe_2_O_3_@TiO_2_ (**162**) and the application of **162** in the synthesis of 1,2,3-triazole derivatives substituted at the 1- and 4-position was described [108]. An aqueous solution of FeCl_3_⋅6H_2_O and FeCl_2_⋅4H_2_O was magnetically stirred in deionized water under an argon atmosphere at room temperature for 5 min. Afterwards, titanium tetrachloride was added to this solution. The pH value was increased to 9 by adding a NH_4_OH solution while stirring at ambient temperature. Then, deionized water was added for dilution of the mixture. After vigorously stirring at 50 °C for 30 min, the magnetic nanoparticles were collected from the solution. The particles were washed with deionized water, crushed, calcinated at 400 °C for 60 min under an argon atmosphere, and then calcinated at 250 °C for 2 h under an oxygen atmosphere to obtain **162**. The material **162** was dispersed in epibromohydrin (E, **158**) using sonication, and this was stirred at 60 °C for 24 h to afford γ-Fe_2_O_3_@TiO_2_–E (**159**). The obtained material was separated using an external magnet and washed with MeOH. Sodium bicarbonate and guanidine hydrochloride (**160**) were added to **159** dispersed in dry toluene. The mixture was heated at reflux for 2 days and then, the guanidinated epibromohydrin-functionalized compound γ-Fe_2_O_3_@TiO_2_–EG (**161**) was magnetically collected, washed with dry dichloromethane, and dried. The material **161** was added to an EtOH solution of Cu(OAc)_2_⋅H_2_O, and this was stirred for 4 h under an argon atmosphere. Cu(II) supported on guanidinated epibromohydrin-functionalized γ-Fe_2_O_3_@TiO_2_, γ-Fe_2_O_3_@TiO_2_–EG–Cu(II) (**162**), was collected, washed with acetone and ethanol, and dried (Scheme 37).

**Scheme 38:**
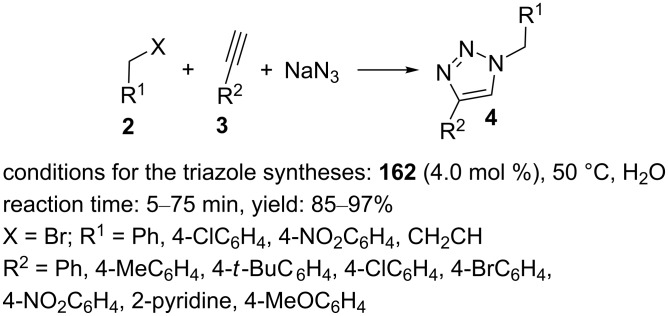
Application of the catalyst **162** in “click” reactions.

The catalyst displayed robust catalytic activity in the synthesis of triazole derivatives via “click” reactions. A variety of terminal alkyl/aryl alkynes was treated with organic halides and sodium azide in water at 50 °C using 4 mol % of the catalyst to generate the corresponding 1,2,3‐triazole products substituted at the 1- and 4-position (Scheme 38).

The magnetic nanocatalyst **162** was easily recycled using an external magnet, and could be reused in six consecutive cycles applying the mentioned reaction conditions, without considerable loss of catalytic activity.

**Scheme 39:**
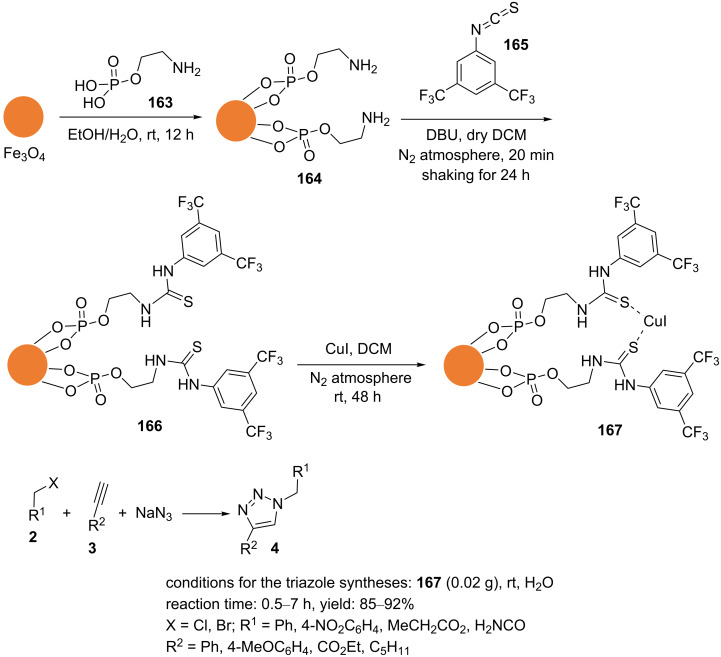
Synthetic route to the catalyst **167** and catalytic application of **167** in “click” reactions.

The Fe_3_O_4_-supported thiourea–copper(I) complex **167** was designed and prepared as a new magnetically separable material [109]. Initially, **164** was created as shown in Scheme 39. In the next step, isothiocyanate **165** and **164** were reacted in dry dichloromethane in the presence of DBU at 0 °C for 20 min. Then, **166** was obtained by shaking the mixture for 24 h. The obtained solid **166** was washed with dry dichloromethane and dried. **166** was dispersed in dry dichloromethane and then, CuI as a copper source was added. After shaking under an argon atmosphere for 2 days, the final material **167** was separated using a magnet and washed with absolute ethanol.

The supported thiourea–copper(I) catalyst **167** exhibited an excellent catalytic activity in the synthesis of triazole derivatives through the “click” reaction of halides, alkynes, and sodium azide in water at room temperature (Scheme 39).

**Scheme 40:**
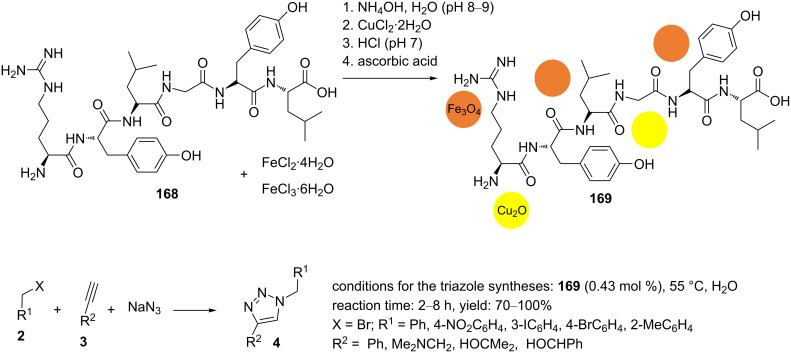
Synthetic route to the catalyst **169** and catalytic application of **169** in “click” reactions.

Shaabani et al. reported magnetic casein as a biosolid to support copper(I) oxide nanoparticles [110]. To immobilize copper(I) oxide nanoparticles on magnetic casein, casein (**168**) was stirred in water for 5 min. In the next step, an aqueous solution of FeCl_2_⋅4H_2_O/FeCl_3_⋅6H_2_O was added to the previous solution, and the reaction mixture was subsequently heated to 80 °C for 1 h. The pH value of the solution was adjusted to 8–9 by the dropwise addition of an aqueous ammonia solution (25%). Casein (**168**), as a biosolid, was dissolved in the previous solution and continuously stirred at 80 °C for 1 h. After cooling, an aqueous solution of CuCl_2_⋅2H_2_O was slowly added to the reaction mixture. Then, an aqueous solution of HCl was used to neutralize the reaction mixture. Subsequently, an aqueous solution of ascorbic acid was added to this. The mixture was heated to 80 °C under continuous stirring for 24 h. The magnetic nanoparticles Cu_2_O/casein@Fe_3_O_4_ (**169**) were collected with a magnet, washed with deionized water and ethanol, and dried (Scheme 40).

The 1,4‐disubstituted 1,2,3‐triazole products were obtained with excellent yields in a regioselective manner using an aqueous medium (Scheme 40). The recyclability of the magnetic catalyst Cu_2_O/casein@Fe_3_O_4_ (**169**) was examined as well. After each cycle, the catalyst was easily recovered, washed with chloroform, and dried before being used in the next cycle. This process was carried out at least three times without any significant decrease in catalytic activity.

**Scheme 41:**
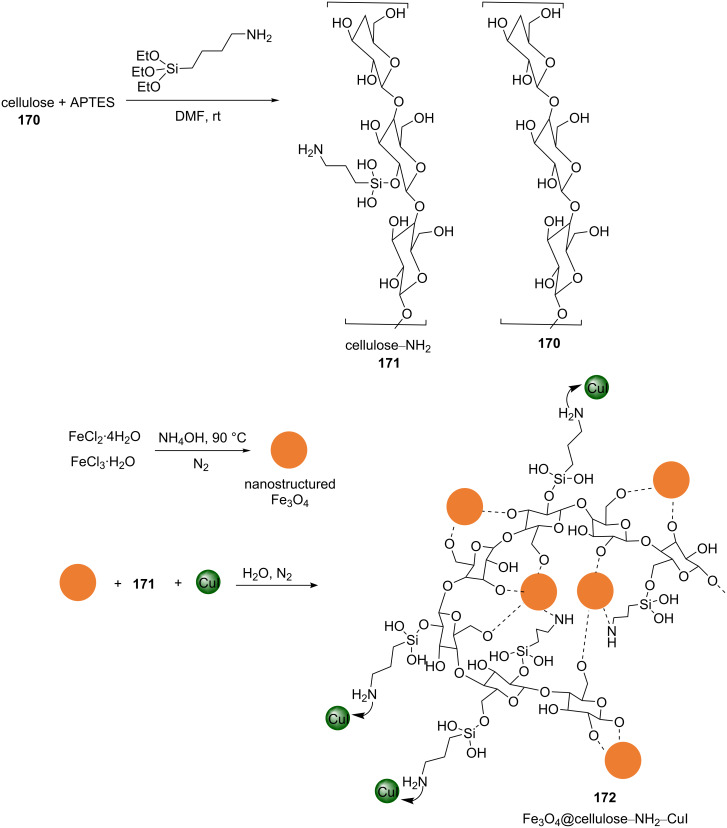
Synthetic route to the catalyst **172**.

Nemati et al. synthesized a modified cellulose‐based nanomagnetite composite to support copper(I) iodide [111]. Cellulose–NH_2_ (**171**) was prepared by the reaction of microcrystals of cellulose (**170**) with APTES in anhydrous dimethylformamide by stirring at room temperature for 2 h. The material **171** was gathered by centrifugation and washed with anhydrous DMF. The magnetic nanoparticles Fe_3_O_4_@cellulose–NH_2_–CuI (**172**) were produced by the addition of an aqueous solution of amino-functionalized cellulose, **171**, to an aqueous mixture of the magnetic Fe_3_O_4_ nanoparticles and copper iodide. The resulting mixture was magnetically stirred under a nitrogen atmosphere at 80 °C for 5 h. The resulting solid **172** was magnetically collected, washed with water, and dried (Scheme 41).

**Scheme 42:**
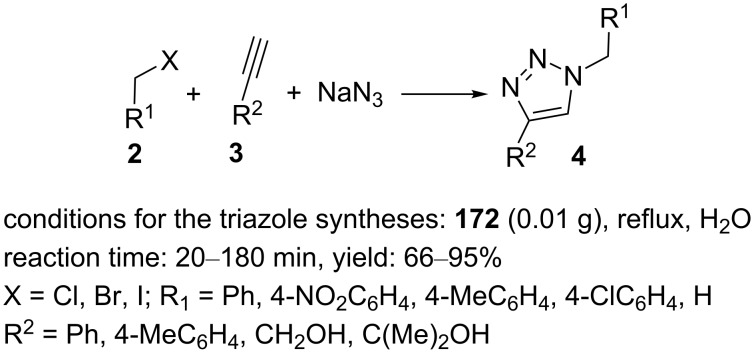
Application of the catalyst **172** in “click” reactions.

The magnetic nanoparticles **172** were applied in the multicomponent reaction of a range of substituted propargylic alcohols or phenylacetylenes, different benzyl halides, and sodium azide to regioselectivity obtain the desired 1,2,3-triazole derivatives substituted at the 1- and 4-positions in an aqueous medium with high yields (Scheme 42).

To determine the reusability of the magnetic ctalyst Fe_3_O_4_@cellulose–NH_2_–CuI (**172**), the compound was used for several cycles in the synthesis of 1-benzyl-4-phenyl-1*H*-1,2,3-triazole derivatives. After the first “click” reaction, the magnetic catalyst was simply recovered with an external magnet and washed with water and ethyl acetate, respectively. The magnetic nanocatalyst could be recycled at least four times with only negligible change in its catalytic activity.

## Conclusion

As emphasized in this review article, a wide variety of functionalized silica/carbon/magnetic material-based supports and their catalytic application in Sharpless–Meldal C–N bond-forming reactions are reported in the literature. The functionalized silica/carbon/magnetic material-based supports demonstrated usefulness in catalysis owing to their diverse structures, chemical/thermal/mechanical stability, facile synthesis, and reusability as well as low toxicity and, in some cases, porous structures and uniform pore sizes. Moreover, from economic and environmental perspectives, these nanocatalysts have advantages over other heterogeneous and homogeneous nanocatalysts. There are a variety of possibilities in this area that require the design and synthesis of ligands and inorganic supports. This review should serve as a starting point for further syntheses of a variety of highly active copper-supporting materials.
